# Effectiveness of fluralaner treatment regimens for the control of canine Chagas disease: A mathematical modeling study

**DOI:** 10.1371/journal.pntd.0011084

**Published:** 2023-01-24

**Authors:** Edem Fiatsonu, Rachel E. Busselman, Gabriel L. Hamer, Sarah A. Hamer, Martial L. Ndeffo-Mbah

**Affiliations:** 1 Department of Veterinary Integrative Biosciences, College of Veterinary Medicine and Biomedical Sciences, Texas A&M University, College Station, Texas, United States of America; 2 Department of Entomology, College of Agriculture and Life Sciences, Texas A&M University, College Station, Texas, United States of America; 3 Department of Epidemiology and Biostatistics, School of Public Health, Texas A&M University, College Station, Texas, United States of America; Universidade Federal de Minas Gerais, BRAZIL

## Abstract

**Background:**

Canine Chagas disease is caused by the protozoan parasite *Trypanosoma cruzi* and transmitted by insect triatomine vectors known as kissing bugs. The agent can cause cardiac damage and long-term heart disease and death in humans, dogs, and other mammals. In laboratory settings, treatment of dogs with systemic insecticides has been shown to be highly efficacious at killing triatomines that feed on treated dogs.

**Method:**

We developed compartmental vector-host models of *T*. *cruzi* transmission between the triatomine and dog population accounting for the impact of seasonality and triatomine migration on disease transmission dynamics. We considered a single vector-host model without seasonality, and model with seasonality, and a spatially coupled model. We used the models to evaluate the effectiveness of the insecticide fluralaner with different durations of treatment regimens for reducing *T*. *cruzi* infection in different transmission settings.

**Results:**

In low and medium transmission settings, our model showed a marginal difference between the 3-month and 6-month regimens for reducing *T*. *cruzi* infection among dogs. The difference increases in the presence of seasonality and triatomine migration from a sylvatic transmission setting. In high transmission settings, the 3-month regimen was substantially more effective in reducing *T*. *cruzi* infections in dogs than the other regimens. Our model showed that increased migration rate reduces fluralaner effectiveness in all treatment regimens, but the relative reduction in effectiveness is minimal during the first years of treatment. However, if an additional 10% or more of triatomines killed by dog treatment were eaten by dogs, treatment could increase *T*. *cruzi* infections in the dog population at least during the first year of treatment.

**Conclusion:**

Our analysis shows that treating all peridomestic dogs every three to six months for at least five years could be an effective measure to reduce *T*. *cruzi* infections in dogs and triatomines in peridomestic transmission settings. However, further studies at the local scale are needed to better understand the potential impact of routine use of fluralaner treatment on increasing dogs’ consumption of dead triatomines.

## Author summary

Chagas disease- caused by the protozoan parasite *Trypanosoma cruzi* and vectored by triatomine insects- poses a serious threat to human and dog health, as infection may go undetected and cause heart disease. Systemic insecticide treatment of dogs is one proposed One Health intervention to reduce *T*. *cruzi* transmission for both dogs and humans, given that dogs are parasite reservoirs. Fluralaner, a systemic insecticide commonly administered to dogs to prevent ectoparasites such as fleas and ticks, induces mortality of triatomines in laboratory settings. We investigated the efficacy of different fluralaner treatment regimens in three endemic peridomestic transmission environments (high, medium, and low) for lowering triatomine density, *T*. *cruzi* infection prevalence, and incidence among dogs using mathematical models. Our simulations suggest that fluralaner treatment is an effective method of reducing *T*. *cruzi* transmission to dogs, and the ideal frequency to treat dogs with fluralaner depends on the transmission environment. Given the intervention may increase the abundance of dead vectors in the dog’s environment, empirical data are needed on the duration of parasite viability within dead vectors and frequency of canine consumption of dead vectors for a more comprehensive evaluation of the intervention.

## Introduction

Chagas disease is a neglected tropical disease that affects approximately 6 million people and is endemic to 21 countries in the Americas [1]. *Trypanosoma cruzi*, the causative agent of Chagas disease, can cause severe cardiac and gastrointestinal disease in humans and other animals [2,3]. It is vectored by triatomine insects (‘kissing bugs’) and is primarily transmitted by infected triatomine fecal material when introduced to a bite wound during or after feeding, or when the infected triatomine or fecal material is consumed [2].

*Trypanosoma cruzi* transmission involves complex interactions between the parasite, multiple host species, and sylvatic vector populations [4,5]. As generalist vectors, triatomines feed on a broad range of domestic and wild mammals and other vertebrate species, each with varying roles in maintaining *T*. *cruzi* transmission cycles [5,6,7,8,9,10]. In domestic cycles of *T*. *cruzi* transmission, triatomines colonize human habitations and feed primarily on humans and domestic animals, while in the sylvatic cycle of transmission, triatomines live in nests or burrows, feeding on diverse wildlife species. In the peridomestic environment- characterized by man-made or natural structures near both human dwellings and natural habitats- *T*. *cruzi* transmission is maintained by populations of triatomines that feed on species often associated with such dwellings, like dogs [11].

The domestic transmission setting has been heavily studied in Latin America, where several local and abundant triatomine species are considered domesticated and frequently colonize homes [12,13,14]. In the southern United States, the triatomine species are considered primarily sylvatic, with an increasing awareness of their impact in peridomestic settings [15,16,17].

In the peridomestic environment, dogs are key bloodmeal hosts for triatomines and serve as competent hosts for *T*. *cruzi* transmission [18,19,20]. Throughout the southern United States, domestic, service, hunting, and government working dog populations have a high prevalence and risk of infection with *T*. *cruzi*. Studies have shown that *T*. *cruzi* prevalence in kennel or shelter environments across Texas, Oklahoma, and Louisiana range from 3.6%-70.1% of dogs infected [19,21,22,23]. Dogs with Chagas disease can develop acute cardiac abnormalities associated with long-term cardiac damage or death, or may remain asymptomatic for years [24]. Prevention of disease is focused on reducing canine exposure to the vectors.

Insecticides play a key role in efforts to control triatomine populations and have been primarily employed as residual sprays in domestic and peridomestic environments [14,25]. Given the concern about insecticide resistance from widespread treatments of homes and the peridomestic environment, systemic insecticides (a.k.a. ectoparasiticides and xenointoxication), which rely on the application of insecticides directly to domestic animals to target triatomines, emerged as an alternative strategy to consider [26]. Multiple studies have now investigated systemic insecticides as a method of using domestic hosts to control triatomine populations and reduce the risk of human *T*. *cruzi* infection [27,28,29,30]. These insecticides, given or applied to a dog, expose any triatomine that feeds on the dog to the insecticide. Bravecto (fluralaner), an oral systemic insecticide treatment given to dogs, inhibits GABA-gated chloride channels and L-glutamate-gated chloride channels in the nervous system of affected insects [31]. It induces nearly 100% mortality in *Triatoma infestans* and *Rhodnius prolixus-* two key triatomine species in *T*. *cruzi* transmission- within a few days of feeding on treated blood and, uniquely, continues killing triatomines up to 7 months after treatment [28,29,32,33]. Fluralaner has also been successfully deployed in the field, significantly reducing infestations and abundance of *T*. *infestans* in the domestic transmission cycle [30]. Further, a prior mathematical modeling study based on the Ross-McDonald malaria model showed that annual treatment of dogs with fluralaner may be impactful in reducing infection rates of dogs in high transmission settings, but may be detrimental in low transmission settings when dog consumption of insects increases following xenointoxication [34]. Targeting dog populations with systemic insecticides in a peridomestic transmission setting may provide a method of triatomine control in areas where dogs are encountering triatomines, which may lead to a reduction in the risk of *T*. *cruzi* infection to dogs, thereby protecting canine health.

For decades, mathematical modeling has been utilized to improve our understanding of *T*. *cruzi* transmission dynamics, often in domestic settings with an emphasis on protecting human health [35,36,37,38,39,40,41,42]. Such efforts have identified reducing domestic vectorial transmission as key to reducing the incidence of Chagas disease in humans [43,44]. Further, housing animals- including dogs- in homes has been linked to an increased risk of *T*. *cruzi* infection in humans [4,45,46,47,48,49,50].

Models investigating sylvatic transmission cycles have been developed to incorporate the multiple sylvatic hosts available, advancing our understanding of interactions between hosts, vectors, and *T*. *cruzi* and highlighting both vector-fecal and oral transmission pathways [42,51,52,53,54,55,56,57]. In peridomestic settings where dogs are the main hosts, host-targeted interventions may provide a valuable tool to reduce triatomine populations and canine exposure to *T*. *cruzi*. Mathematical modeling can provide insight into the peridomestic cycle of *T*. *cruzi-* transmission and the potential population-level effects of host-targeted interventions, specifically when dogs are the primary host.

In this study, we developed a series of compartmental models to evaluate transmission dynamics between triatomines and dogs in the peridomestic environment, considering the seasonality of triatomine vectors, varying prevalence of *T*. *cruzi* infections in dogs and triatomines, and the potential impact of triatomine migration between peridomestic and sylvatic transmission settings. We then used our models to evaluate the effectiveness of different treatment regimens of the systemic insecticide fluralaner for the control of reducing triatomine populations and *T*. *cruzi* infections. Based on the findings in Rokhsar et al. [34], we also consider the potential impact of increased consumption of triatomines and oral transmission when fluralaner is routinely given to dogs.

## Methods

### Model structure

We developed three compartmental vector-host SI (Susceptible-Infected) models for the transmission of *T*. *cruzi* between triatomines and dogs. The models are: (1) a baseline model that does not account for the impact of seasonality on triatomine dynamics, (2) a seasonality model that explicitly accounts for the impact of seasonality on triatomine dynamics, and (3) a spatially coupled model accounting for the movement of triatomine between peridomestic and sylvatic habitats. We used these models to evaluate the population-level effectiveness of different dog systemic insecticide treatment regimens on *T*. *cruzi* prevalence in dogs and triatomine populations in different *T*. *cruzi* transmission settings.

### Single vector-host population model

**Fig 1:**
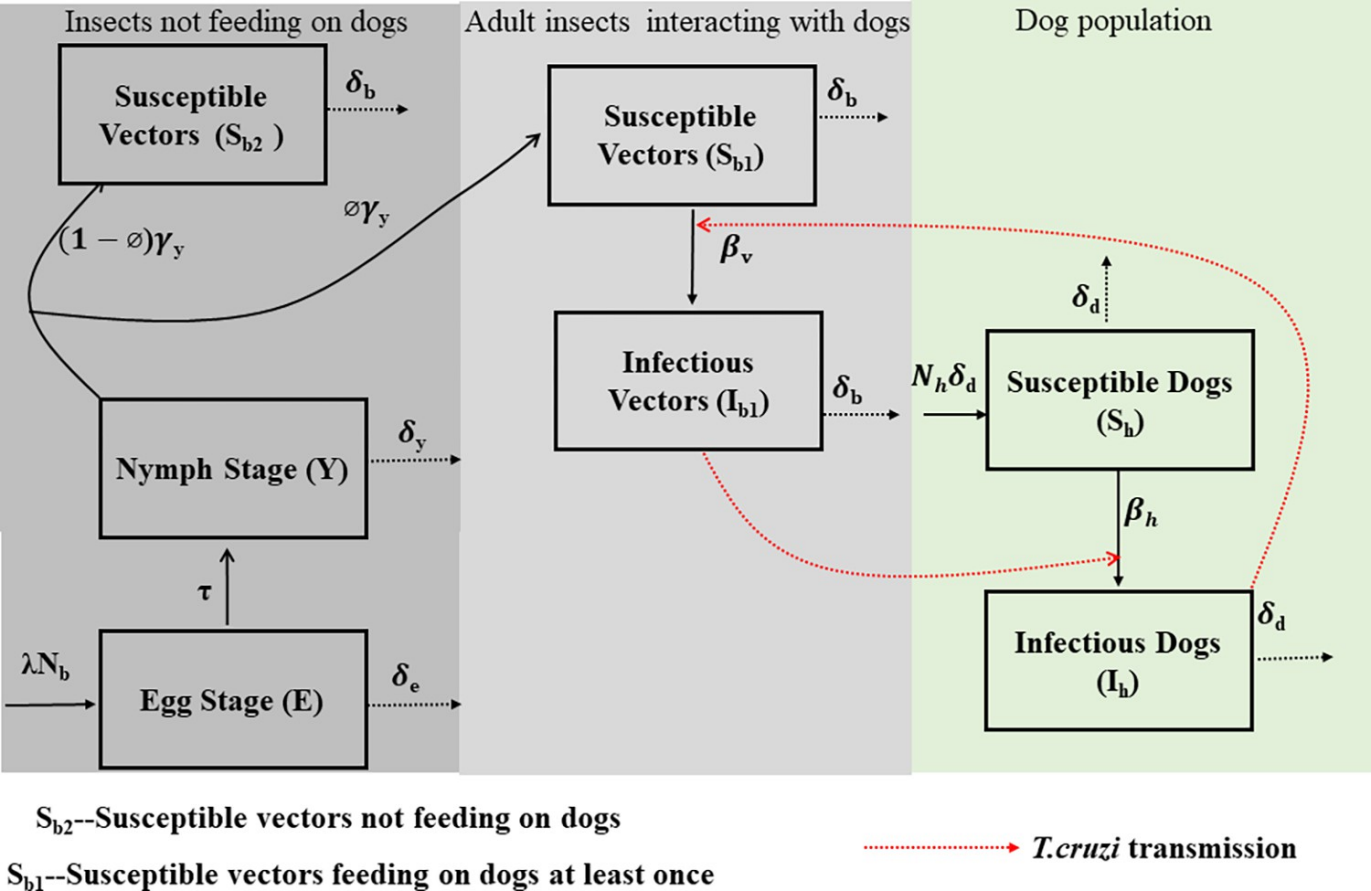
Single vector-host population model structure.

Here, we consider a model with a single vector-host population for the transmission of *T*. *cruzi* between triatomines and peridomestic dogs. Like most Chagas disease models [35,43,45,46,51,58], here we ignore the impact of seasonality on triatomine dynamics by assuming all model parameters to be constant over time. We used an age-structured model for the triatomine population (Fig 1). The age structure includes egg stage, nymph, and adult stage. We assume that eggs (E) are laid at a per-female reproduction rate λ, and hatch into nymphs (Y) at the rate τ. We assume the surviving rate from egg to nymph to be density-dependent with an environmental carrying capacity κ. Nymphs molt into adults at maturation rate *γ*_*y*_. We assume no vertical transmission of *T*. *cruzi* from adult triatomines to their offspring, which is supported by past studies [59,60]. In addition, we assume that nymphs remain uninfected until they molt into full-grown adults with fully developed wings to be able to disperse in search of blood meals. This assumption is predicated on the fact that in our model triatomine can only get infected by feeding on infected dogs, and nymphs are unlikely to feed on dogs.

In this transmission setting, we assume that dogs are the main/only competent host for *T*. *cruzi* and that a small proportion of adult triatomines will never feed on dogs but instead will feed on other incompetent hosts (which are not modeled explicitly). These triatomines will remain uninfected with *T*. *cruzi*, and we denote them as *S*_*b*2_. The remaining proportion of adult triatomines, which feed on dogs at least once, is denoted using the subscript _*b*1_. Here, susceptible adult triatomines, *S*_*b*1_, can become infected, *I*_*b*1_, at a transmission rate *β*_*v*_. The population density of triatomines feeding on dogs is equal to *N*_*b*1_ = *S*_*b*1_+*I*_*b*1_. However, the total population density of adult triatomines is equal to *N*_*b*_ = *S*_*b*1_+*I*_*b*1_+*S*_*b*2_. For simplicity, we assume the dog population, *N*_*h*_, to be constant, where the birth rate equals the death rate. Each dog could be in either mutually exclusive disease state: susceptible, *S*_*h*_ (not infected with *T*. *cruzi* and able to become infected) and infectious, *I*_*h*_ (infected with *T*. *cruzi* and able to transmit). *T*. *cruzi* transmission from infected dogs to triatomines occurs at a transmission rate *β*_*h*_. We assume that the transmission rate *β*_*h*_ accounts for two contact-based infection processes: stercorarian (vector fecal contamination) and oral. Here, dogs can be infected with *T*. *cruzi* either through the feces of infected triatomines or by oral consumption of infected triatomines. Natural mortality rates of dogs, adult triatomines, nymphs, and eggs are respectively denoted by *δ*_*d*_, *δ*_*b*_, *δ*_*y*_, and *δ*_*e*_. The dynamics of *T*. *cruzi* transmission between triatomines and dogs are described by a system of ordinary differential equations denoted as Model 1 (see Fig 1 for a schematic description of the model).

Model 1

$$\frac{dE}{dt}=\lambda N_{b}-\tau E-\delta_{e}E$$ (1a)

$$\frac{dY}{dt}=\tau E(1-\frac{Y+N_{b}}{\kappa })-\gamma_{y}Y-\delta_{y}Y$$ (1b)

$$\frac{dS_{b1}}{dt}=\phi \gamma_{y}Y-\beta_{v}S_{b1}\frac{I_{h}}{N_{h}}-\delta_{b}S_{b1}$$ (1c)

$$\frac{dI_{b1}}{dt}=\beta_{v}S_{b1}\frac{I_{h}}{N_{h}}-\delta_{b}I_{b1}$$ (1d)

$$\frac{dS_{b2}}{dt}=(1-\phi {)\gamma }_{y}Y-\delta_{b}S_{b2}$$ (1e)

$$\frac{dS_{h}}{dt}=\delta_{d}N_{h}-\beta_{h}S_{h}\frac{I_{b1}}{N_{b1}}-\delta_{d}S_{h}$$ (1f)

$$\frac{dI_{h}}{dt}=\beta_{h}S_{h}\frac{I_{b1}}{N_{b1}}-\delta_{d}I_{h}$$ (1g)

Model’s parameters are described in Table 1. All parameter values are obtained from published literature with the exception of the proportion of the triatomines that would feed on dogs (ϕ), and the carrying capacity (κ).

**Table 1:** Key model input parameters, values, and sources.

Parameter	Symbol	Value	Source
Proportion of adult triatomines feeding at least once on dogs	ϕ	0.95	[61]
Carrying capacity	κ	37018.vec/km^2^	Estimated
Triatomine per-female egg production	λ	475/yr	[62,63,64]
Egg hatching rate	τ	23.7/yr	[65,66]
Nymph maturity rate	γ_y_	1.73/yr	[66,67]
Egg mortality rate	δ_e_	0.36/yr	[62]
Nymph mortality rate	δ_y_	1.46/yr	[66,68]
Adult triatomine mortality rate	δ_b_	0.56/yr	[51,68]
Triatomine population density	*N*_*b*_	31600.vec/km^2^	[51]
Probability of vector infection per bite on infectious dog	ρ	0.3082	[69]
Dog population density	*N*_*h*_	1000.host/km^2^	Estimated from [70,71]
Dog mortality rate	δ_d_	0.1/yr	[43]

Because canine Chagas disease is endemic in many countries in the Americas including the southern part of the United States, we reasonably assume that our system is at equilibrium. We use * to demote state variables at equilibrium with *N*_*b*_ = *S*_*b*1_*+*S*_*b*2_*+*I*_*b*1_*. The carrying capacity κ, is estimated at the steady state (equilibrium) as:

We first find the equilibrium value for egg and the nymph stage.

Egg stage equilibrium:

$$\frac{dE}{dt}=\lambda N_{b}-\tau E-\delta_{e}E=0,$$

$$\lambda N_{b}=E(\tau +\delta_{e}),$$

$$E^{*}=\frac{\lambda N_{b}}{\left(\tau +\delta_{e}\right)}.$$

Nymph stage at equilibrium:

$$\frac{dY}{dt}=\tau E(1-\frac{Y+N_{b}}{\kappa })-\gamma_{y}Y-\delta_{y}Y=0,$$

$$\tau E(1-\frac{N_{b}}{\kappa })=Y[(\gamma_{y}+\delta_{y})+\frac{\tau E}{\kappa }],$$

$$Y^{*}=\frac{\tau \lambda N_{b}(\kappa -N_{b})}{\kappa (\delta_{e}+\tau )(\gamma_{y}+\delta_{y})+\tau \lambda N_{b}},$$

Now, we sum the differential equations for adults triatomine and set the results into zero.

Thus, $\frac{{dN}_{b}}{dt}=\frac{dS_{b1}}{dt}+\frac{dS_{b2}}{dt}+\frac{dI_{b1}}{dt}=0,$

$$\frac{{dN}_{b}}{dt}=\gamma_{y}Y^{*}-\delta_{b}N_{b}=0,$$

$$N_{b}=\frac{\gamma_{y}Y^{*}}{\delta_{b}},$$

$$N_{b}=\frac{\gamma_{y}}{\delta_{b}}\left[\frac{N_{b}\lambda \tau (\kappa -N_{b})}{\kappa (\delta_{e}+\tau )(\gamma_{y}+\delta_{y})+\lambda \tau N_{b}}\right],$$

$$\kappa =\frac{\tau \lambda N_{b}(\delta_{b}+\gamma_{y}N_{b})}{\tau \lambda \gamma_{y}N_{b}-\delta_{b}(\delta_{e}+\tau )(\gamma_{y}+\delta_{y})}$$

The parameters *δ*_*e*_, *δ*_*y*_, *δ*_*b*_ are the egg, nymph, and adult mortality rate, respectively. We assumed ϕ to be equal to 0.95 and tested the robustness of our results for ϕ greater or equal to 0.9.

We have *S*_*b*1_* = *N*_*b*_−*S*_*b*2_*−*I*_*b*1_* and defined disease prevalences at equilibrium as $\frac{{I_{b1}}^{*}}{N_{b}}=\frac{ib\times N_{b}}{N_{b}}=y_{v}$ and $\frac{{I^{*}}_{h}}{N_{h}}=\frac{id\times N_{h}}{N_{h}}=y_{h}$, where *ib*, *id* are empirical estimates of *T*. *cruzi* infection prevalence in triatomines and dogs respectively. The proportion of *S*_*b*1_ and *S*_*b*2_ at equilibrium state are also defined as $s_{b1}=\frac{{S_{b1}}^{*}}{N_{b}}$, and $s_{b2}=\frac{{S_{b2}}^{*}}{N_{b}}$. The steady state of Model 1 (setting the equilibrium equations for Eqs 1D and 1G into zero) can be written as:

$$\beta_{v}(1-y_{v}-s_{b2})y_{h}-\delta_{b}y_{v}=0,\beta_{h}(1-y_{h})y_{v}-\delta_{d}y_{h}=0.$$

We can now solve these equations for the transmission rates *β*_*h*_ and *β*_*v*_ as done in [51]. The corresponding transmission rates are therefore computed as:

$$\beta_{v}=\frac{\delta_{b}y_{v}}{(1-y_{v}-s_{b2})y_{h}}\;\mathrm{and}\;\beta_{h}=\frac{\delta_{d}y_{h}}{(1-y_{h})y_{v}}.$$

We considered three transmission settings (high, medium, and low) and estimated the corresponding transmission rates for each setting (Table 2).

**Table 2:** *Trypanosoma cruzi* infection prevalence and transmission rates.

Description	Variable	High	Medium	Low
Dog prevalence	*id*	0.30	0.15	0.08
Triatomine prevalence	*ib*	0.56	0.25	0.13
Annual host transmission rate (1/year)	β_h_	0.0745	0.0687	0.0651
Annual vector transmission rate (1/year)	*β*_*v*_	2.5351	1.2941	1.0823

The values of dog and insect prevalence are informed from [18,72,73,74,75].

### Single vector-host population model with seasonality

Here, we extend our SI vector-host model (Model 1) to capture the impact of seasonality on the triatomine life cycle and *T*. *cruzi* transmission dynamics. Specifically, we assume seasonality patterns of nymphal maturation rate and transmission rate (contact rate between triatomines and dogs driven by triatomine seasonal dispersal in search of blood meal [76]). We denote this model as Model 2.

We assume that seasonality in nymphal maturation rate (nymphs molting into adults) follows a stepwise function with high activities during spring, lower activities during summer and fall, and none during winter. We define the maturation rate as

$$\gamma_{y}(t)=\gamma_{y}^{0}g(t,\varepsilon )\;with\;\int_{0}^{1}g(t,\varepsilon )dt=1.$$

where *ε* is the relative activity level of triatomines during summer and fall compared to spring and set to be equal to 0.25 (we also consider *ε* = 0.5), and $\gamma_{y}^{0}$ is the annual maturation rate in the absence of seasonality (Model 1).

The contact rate between triatomines and dogs, *h*(*t*), was defined as a piecewise function whose values were informed from empirical data of triatomine host biting over a one-year period [76]. We defined the transmission rates as follow:

$$\beta_{v}(t)={\beta_{v}}^{0}h(t)\;and\;\beta_{h}(t)={\beta_{h}}^{0}h(t)$$

with $\int_{0}^{1}h(t)=1$, where *β*_*v*_^0^ and *β*_*h*_^0^ are annual transmission rates in the absence of seasonality (Model 1).

### Spatially coupled vector-host model with seasonality

Here, we consider a spatially coupled vector-host model for the spread of *T*. *cruzi* between peridomestic and sylvatic transmission cycles. In the peridomestic setting, dogs are assumed to be the main/only competent reservoir host for *T*. *cruzi* transmission. In the sylvatic setting, we assume diverse wildlife reservoir species competent for *T*. *cruzi* transmission [e.g. raccoon (*Procyon lotor)*, opossum (*Didelphis virginiana*), woodrat (*Neotoma* spp.)] and all adult triatomines are likely to feed on at least one *T*. *cruzi-*competent host. For simplicity, we aggregated the wildlife *T*. *cruzi-*competent species into a single host population. In both settings, we assume that triatomines have the same seasonality behavior described in Model 2. So, we have a vector-host SI model for *T*. *cruzi* transmission in the peridomestic and sylvatic transmission settings. We assume the host populations to be constant in each setting and only adult triatomines move between the two settings, with *η*(*t*) being the movement rate of adult bugs from peridomestic to sylvatic habitat and *ξ*(*t*) being the movement rate from sylvatic to peridomestic habitat. The movement rates are defined as *η*(*t*) = *η*^0^*m*(*t*) and *ξ*(*t*) = *ξ*^0^(*t*)*m*(*t*) with $\int_{0}^{1}m(t)dt=1$, where *η*^0^ and *ξ*^0^ are the average annual movement rates and *m*(*t*) is the seasonal behavior function. *m*(*t*) was designed as a piecewise function whose values were informed from empirical data on adult triatomine dispersal over time [77]. The system of equations of this model (Model 3) is provided in S1 Text.

### Dog treatment

**Fig 2:**
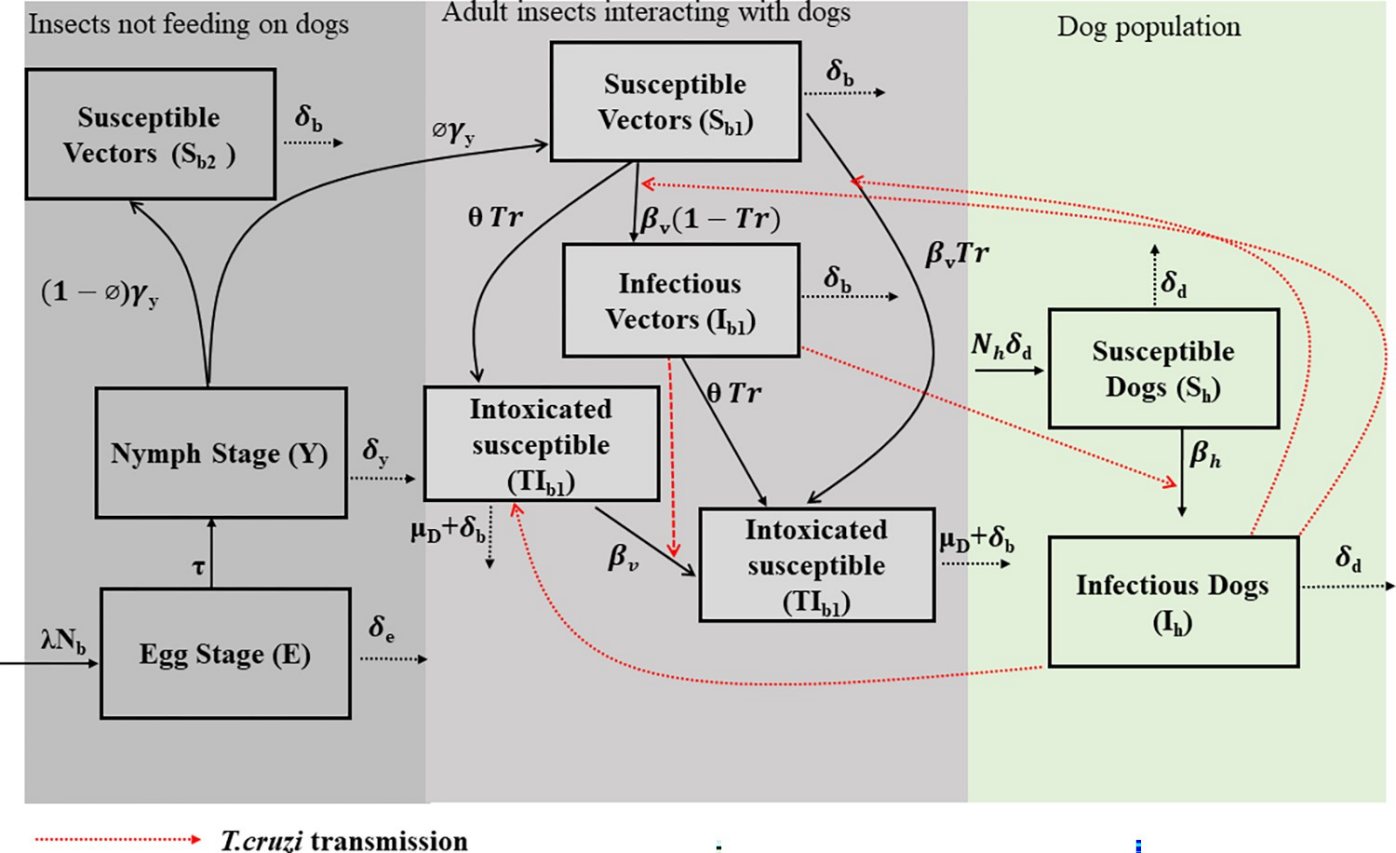
Model structure with treatment. *TS*_*b*1_ and *TI*_*b*1_ is the density of susceptible and infected adult bugs that feed on fluralaner-treated dogs and will die from fluralaner intoxication.

Fluralaner, an oral systemic insecticide, is used in dogs to prevent tick and flea infestations [32,78,79]. In this study, we evaluated the effectiveness of fluralaner treatment against *T*. *cruzi* infection in dogs and triatomines. We focused on fluralaner as the systemic insecticide because of the availability of empirical efficacy data on its ability to kill triatomines. These data, obtained from a systematic laboratory study [32], provide estimates of the monthly efficacy of fluralaner on killing triatomines that fed on dogs during the first twelve months following dogs’ treatment, as well as the mortality rate of those triatomines (duration from feeding to death at an hourly rate) [32]. The results from this study were used to inform treatment efficacy (*Tr*) and treatment-induced mortality rate (*μ*_*d*_) in our model (Fig 2). Although the reference laboratory study used *Triatoma infestans*- a species native to South America- we recently evaluated the ability of fluralaner-treated dogs to kill *Triatoma gerstaeckeri* nymphs that were the progeny of wild-caught adults from South Texas, with similar results [80].

To compute the number of susceptible adult bugs that have fed on treated dogs, we derived contact rate between adult bugs and dogs as *θ* = *β*_*v*_/*ρ* where *ρ* is the probability of adult bug infection per instance fed on infected dog and *β*_*v*_ is the transmission rate from infected dogs to adult triatomine.

### Treatment strategies

We consider four fluralaner treatment regimens: 3-month (with dogs treated once every three months), 6-month, 9-month, and 12-month. For each regimen, treatment efficacy and induced mortality rate were informed by empirical data [32]. We used our models to evaluate the effectiveness of each regimen for reducing *T*. *cruzi* infection prevalence and incidence among dogs and adult triatomines, as well as adult triatomine density in three transmission settings (high, medium, and low: Table 2), with and without seasonal triatomine transmission behavior and spatial coupling. In the spatially coupled model, we assume that only peridomestic dogs received treatment. We compare the predicted effectiveness of the four regimens to identify the most effective regimen in each setting. Our effectiveness outcomes are: the reduction of *T*. *cruzi* prevalence in dogs, reduction of adult triatomine density, reduction of *T*. *cruzi* incidence on triatomines and dogs, and cumulative dog and triatomine infections averted.

### Impact of increased consumption of dead triatomines

Dog treatment with fluralaner will likely result in substantial increase of triatomine mortality, but the degree to which this will result in increased consumption by dogs of dead insects is not known. Rokhsar et al [34] assumed that dogs consumed 80% of bugs killed by treatment and that consumption happened immediately upon death. However, given parasites may not remain viable in dead insects especially those that are exposed to the hot and dry ambient conditions of Chagas endemic regions, and given no data to suggest a high level of insectivory by dogs, we primarily conduct our analysis assuming that dog treatment does not result in a significant increase of triatomine consumption by dogs. We further extend our analysis by investigating the potential impact of increased consumption of dead bugs on the effectiveness of treatment regimens for reducing *T*. *cruzi* infection prevalence in dogs. We assume that infected bugs can remain infectious on average two days after death. The probability of oral infection per infected bug consumed is 0.177 (17.7%) [51].

## Results

### Base model

With no treatment strategies added to our model, the prevalence of *T*. *cruzi* in triatomines ranged from 56.0%-13.0%, and infection prevalence in dogs ranged from 30%-8% in the high to low transmission settings, respectively (Table 2). We computed the corresponding annual transmission rates for triatomines and dogs in each transmission setting (Table 2). We used our model to evaluate the effectiveness of systemic insecticide treatment of dogs with fluralaner for the control of *T*. *cruzi* transmission. Additionally, we consider the effects of increased dog consumption of triatomines, thus an increased oral transmission rate, when dogs are given fluralaner.

### Single vector-host model without seasonality

**Fig 3:**
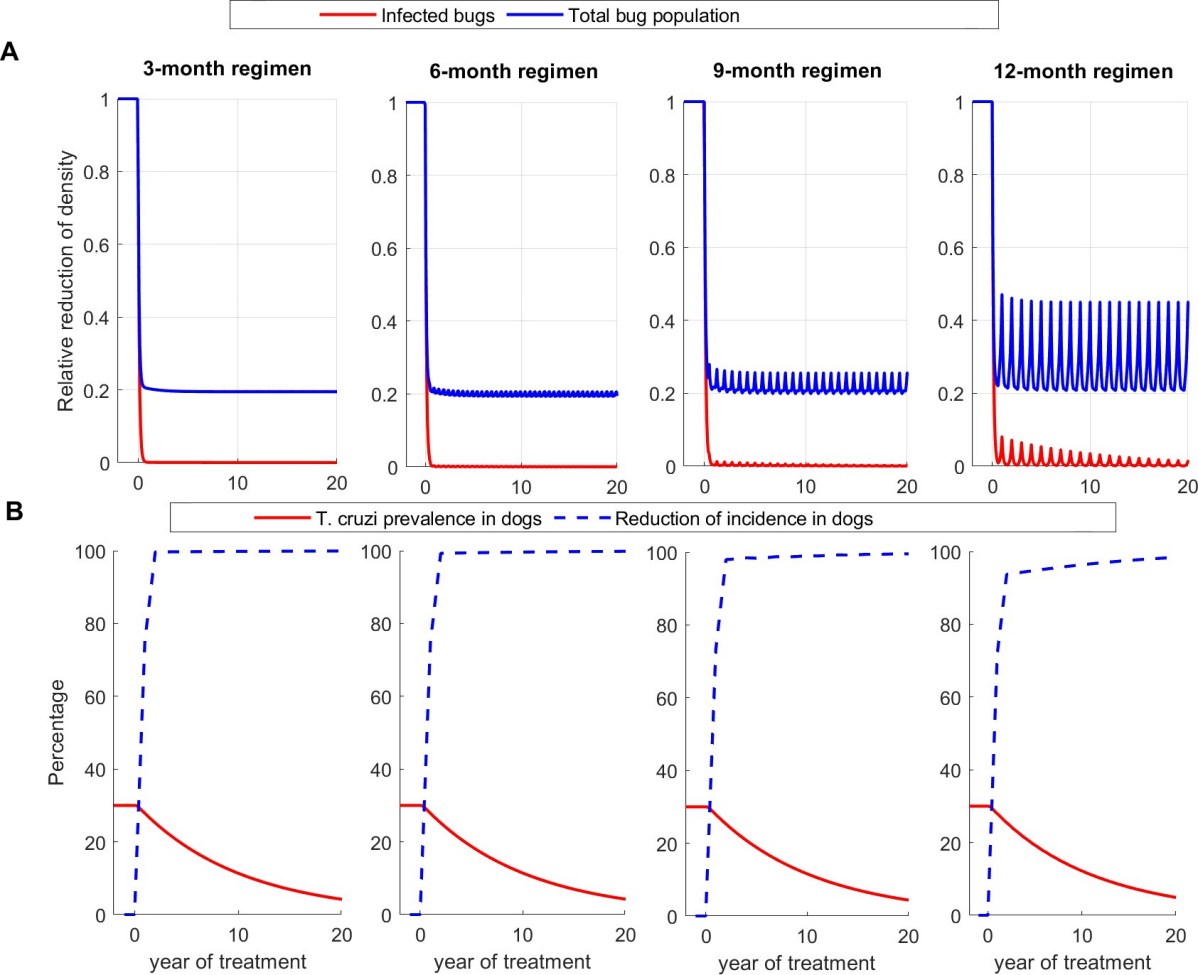
Effectiveness of systemic insecticide treatment of dogs with fluralaner for the control of canine Chagas in a high transmission setting with 3-month, 6-month, 9-month, and 12-month treatment regimens using Model 1. (A) Reduction of total population density (blue) and *T*. *cruzi* infections in triatomines (red), (B) Reduction of *T*. *cruzi* infection prevalence (red) and incidence in dogs (blue). Effectiveness is evaluated using the single vector-host model without seasonality.

**Fig 4:**
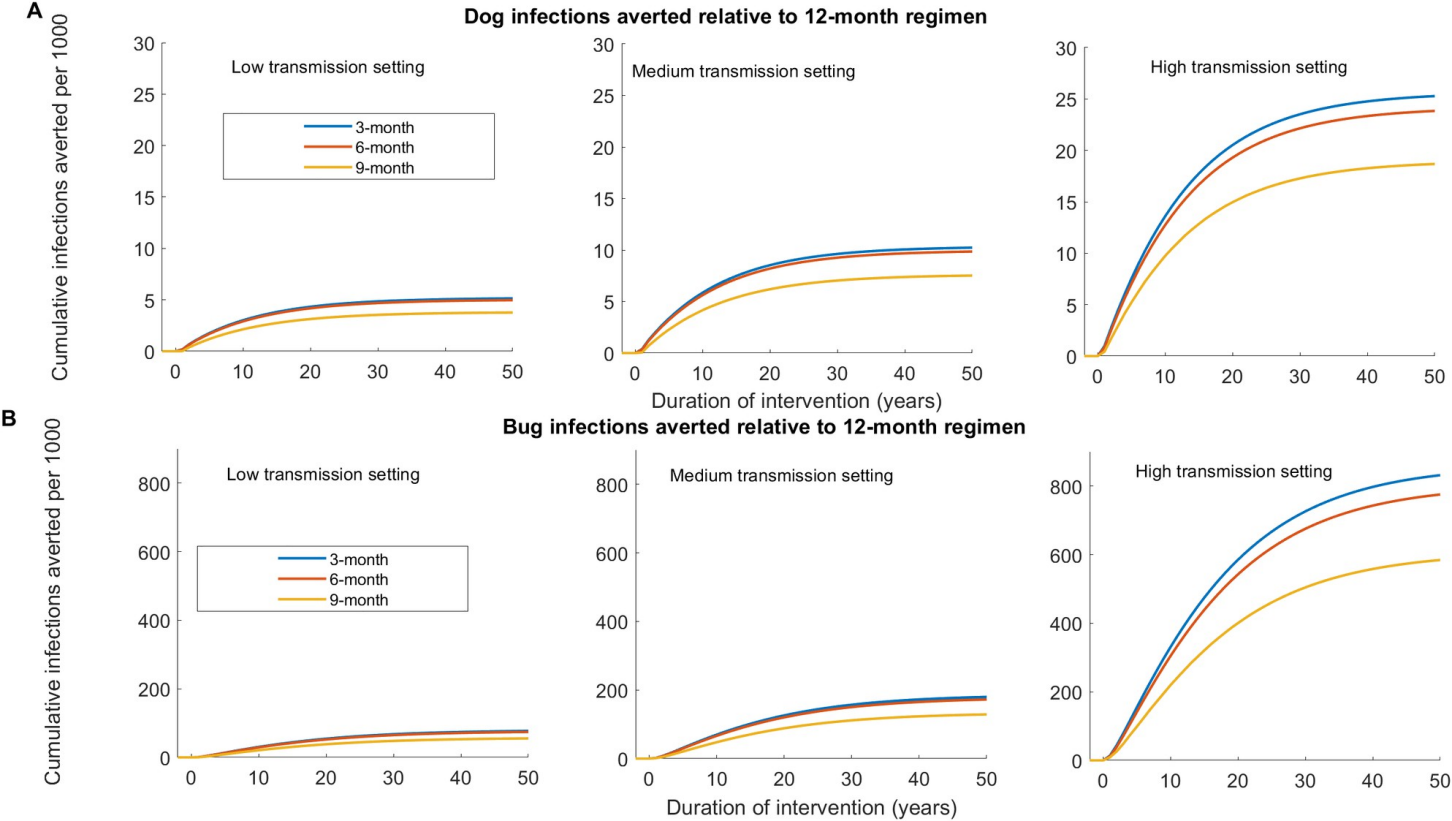
Relative effectiveness of dog treatment regimen for reducing *T*. ***cruzi* infections among dogs and triatomines compared to a 12-month treatment regimen using Model 1.** Effectiveness is computed using the single host-vector model without seasonality. (A) Cumulative additional dog infections averted under the 3-month, 6-month, and 9-month regimen relative to the 12-month regimen in the low, medium, and high transmission settings. (B) Cumulative additional triatomine infections averted under the 3-month, 6-month, and 9-month regimen relative to the 12-month regimen in the low, medium, and high transmission settings.

To evaluate the effectiveness of the treatment regimens, we first use a standard vector-host model for *T*. *cruzi* transmission between dogs and triatomines that does not account for the impact of seasonality on triatomine activity. In all transmission settings, we observed a prompt and substantial decline in vector population density, vector *T*. *cruzi* prevalence, and dog *T*. *cruzi* incidence following the initiation of treatment under all regimens (Figs 3, A, and B in S1 Text). Results for the high transmission setting are shown in Fig 3, and those of low and medium transmission settings are shown in Figs A and B in S1 Text, respectively. For example, after 10 years of treatment in a high transmission setting, triatomine population density was reduced on average by 80.4%, 75.8%, 74.4% and 66.4% under the 3-month, 6-month, 9-month, and 12-month treatment regimen, respectively (Fig 3A), whereas triatomine *T*. *cruzi* prevalence and dog *T*. *cruzi* incidence were reduced on average by more than 98% and 96%, respectively (Fig 3). The effectiveness of treatment regimens for reducing dog infection was shown to increase with transmission intensity; with high transmission setting having the highest reduction and low transmission having the lowest reduction (Fig 4 and Table A in S1 Text). For example, after five years of treatment, *T*. *cruzi* prevalence among dogs was reduced by 37.7%, 37.5%, 37.1%, and 35.7% under the 3-month, 6-month, 9-month, and 12-month treatment regimen, respectively, and by 63.1%, 62.0%, 61.4% and 59.4% after 10 years (Table A in S1 Text).

To compare the effectiveness of the treatment regimens, we computed the cumulative *T*. *cruzi* infections averted among dogs and triatomines under 3-month, 6-month, and 9-month regimens relative to the 12-month regimen (Fig 4). In all transmission settings, we showed a marginal difference between the relative effectiveness of the 3-month and 6-month regimens and the 9-month regimen is more effective than 12-month (Fig 4).

### Single vector-host model with seasonality

**Fig 5:**
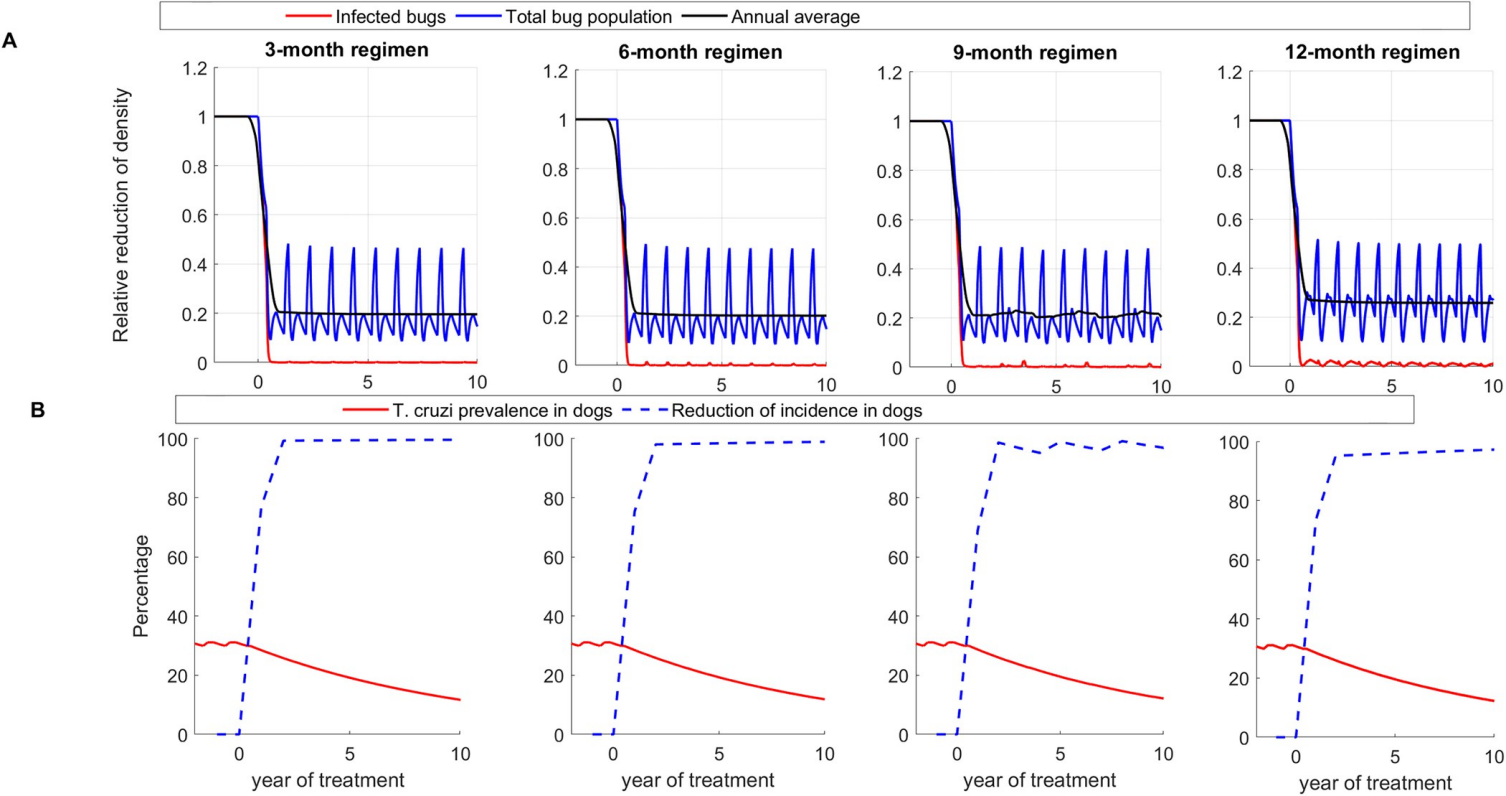
Effectiveness of systemic insecticide treatment of dogs with fluralaner for the control of canine Chagas in a high transmission setting with 3-month, 6-month, 9-month, and 12-month treatment regimens using Model 2. (A) Reduction of total population density (blue) and *T*. *cruzi* infections in triatomines (red), (B) Reduction of *T*. *cruzi* infection prevalence (red) and incidence in dogs (blue). Effectiveness is evaluated using the single vector-host model with seasonality.

**Fig 6:**
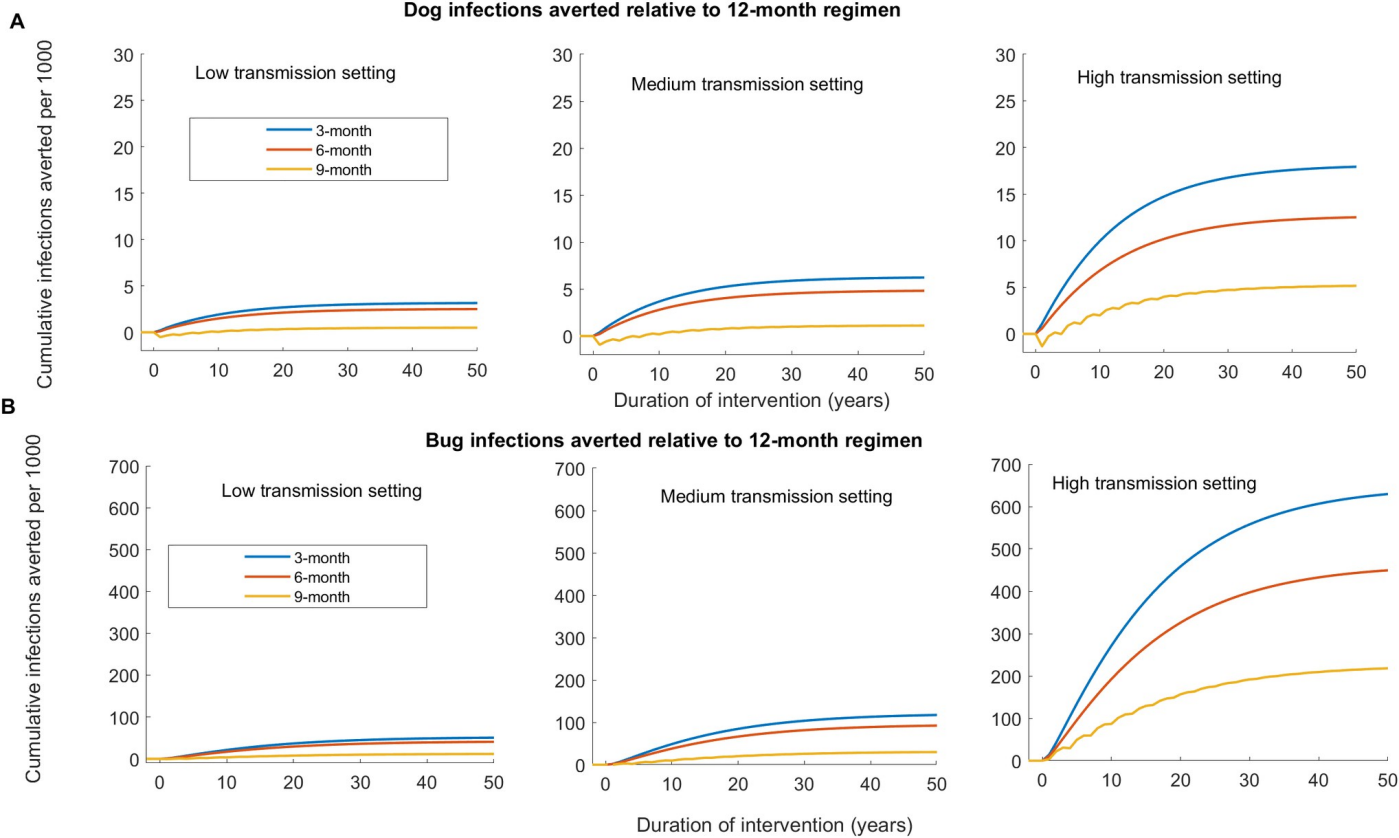
Relative effectiveness of dog treatment regimen for reducing *T*. ***cruzi* infections among dogs and triatomines compared to a 12-month treatment regimen using Model 2.** Effectiveness is computed using the single host-vector model with seasonality. (A) Cumulative additional dog infections averted under the 3-month, 6-month, and 9-month regimen relative to the 12-month regimen in the low, medium, and high transmission settings. (B) Cumulative additional triatomine infections averted under the 3-month, 6-month, and 9-month regimen relative to the 12-month regimen in the low, medium, and high transmission settings.

Similarly, to the no seasonality model, in the model that considers seasonality we observed a rapid reduction in vector population density, *T*. *cruzi* prevalence, and dog *T*. *cruzi* incidence in all transmission settings (Figs 5, C, and D in S1 Text). Results for the high transmission setting are shown in Fig 5, and those of low and medium transmission settings are shown in Figs C and D in S1 Text, respectively. For example, after 10 years of treatment in the high transmission setting, triatomine population density was reduced on average by 80.4%, 79.8%, 78.3%, and 74.1% under the 3-month, 6-month, 9-month, and 12-month treatment regimens, respectively (Fig 5A), whereas triatomine *T*. *cruzi* prevalence and dog *T*. *cruzi* incidence were reduced on average by more than 99% and 97%, respectively (Fig 5). Under the 3-month, 6-month, 9-month, and 12-month treatment regimen, *T*. *cruzi* prevalence among dogs was reduced by 36.2%, 35.7%, 35.0%, and 34.7%, respectively, after five years of repeated treatment, and by 61.1%, 60.5%, 59.6%, and 59.1% after ten years of repeated treatment (Table A in S1 Text). In comparison to the 12-month regimen, relative cumulative *T*. *cruzi* infections averted over the first 20 years of treatment are 15, 11, and 4 per 1000 dogs and 459, 326, and 157 infections per 1000 bugs under the 3-month, 6-month, and 9-month treatment regimens, respectively (Fig 6). In low and medium transmission settings, the 9-month and 12-month treatment regimens were shown to be equally effective, and minimal differences were observed between the 3-month and 6-month regimens (Fig 6). Though our analysis was conducted for *ε*, the relative activity level of triatomines during summer and fall compared to spring, equals to 0.25, similar results were obtained for *ε* = 0.5 (Fig E in S1 Text). The effectiveness of treatment regimens for reducing dog infection was shown to increase with transmission intensity; with high transmission setting having the highest reduction and low transmission having the lowest reduction (Fig 6 and Table A in S1 Text)

### Spatially coupled vector-host model

**Fig 7:**
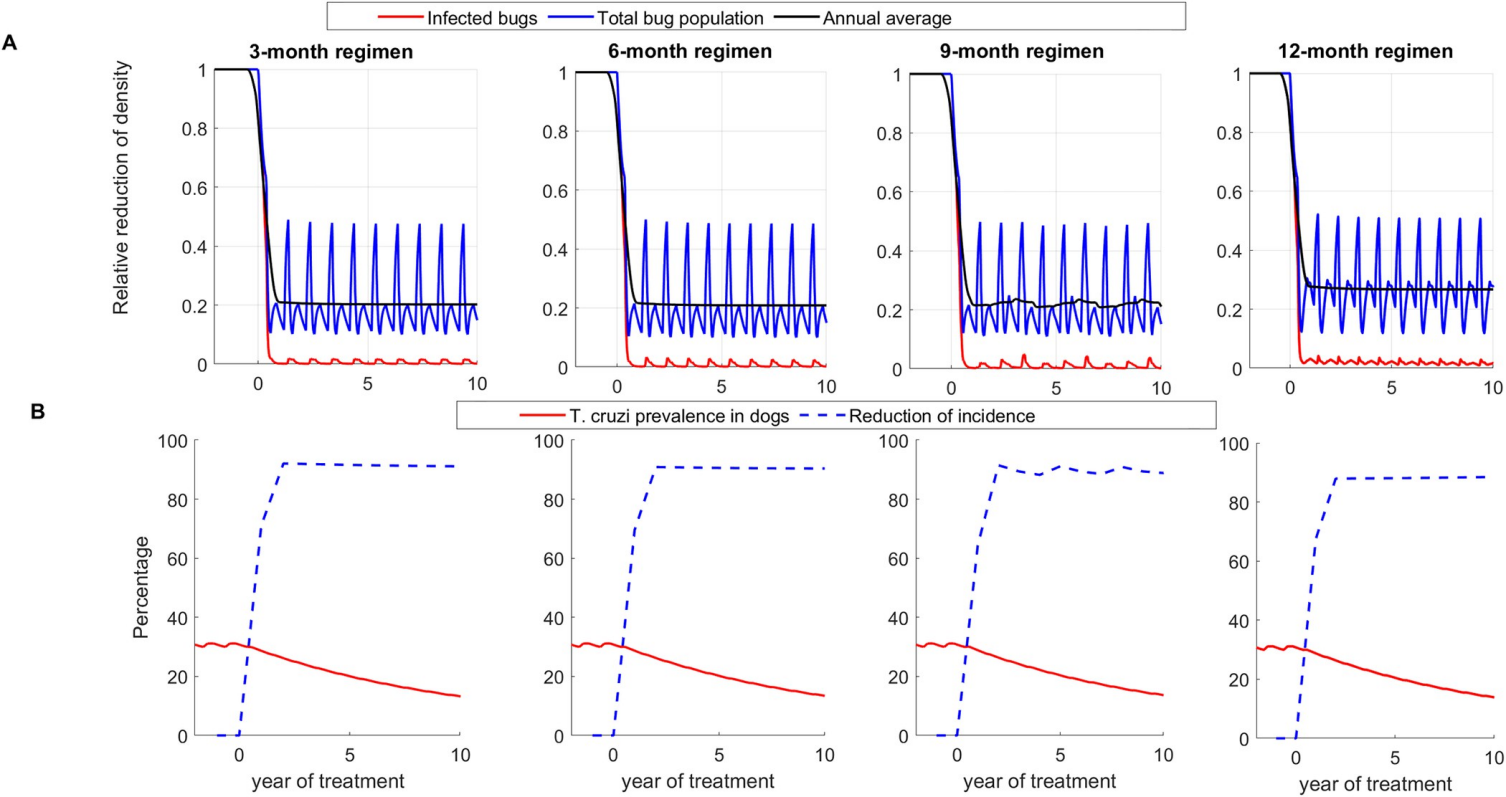
Effectiveness of systemic insecticide treatment of dogs with fluralaner for the control of canine Chagas in a high transmission setting with 3-month, 6-month, 9-month, and 12-month treatment regimens using Model 3. (A) Reduction of total population density (blue) and *T*. *cruzi* infections in triatomines (red), (B) Reduction of *T*. *cruzi* infection prevalence (red) and incidence in dogs (blue). Effectiveness is evaluated using the spatially coupled model.

**Fig 8:**
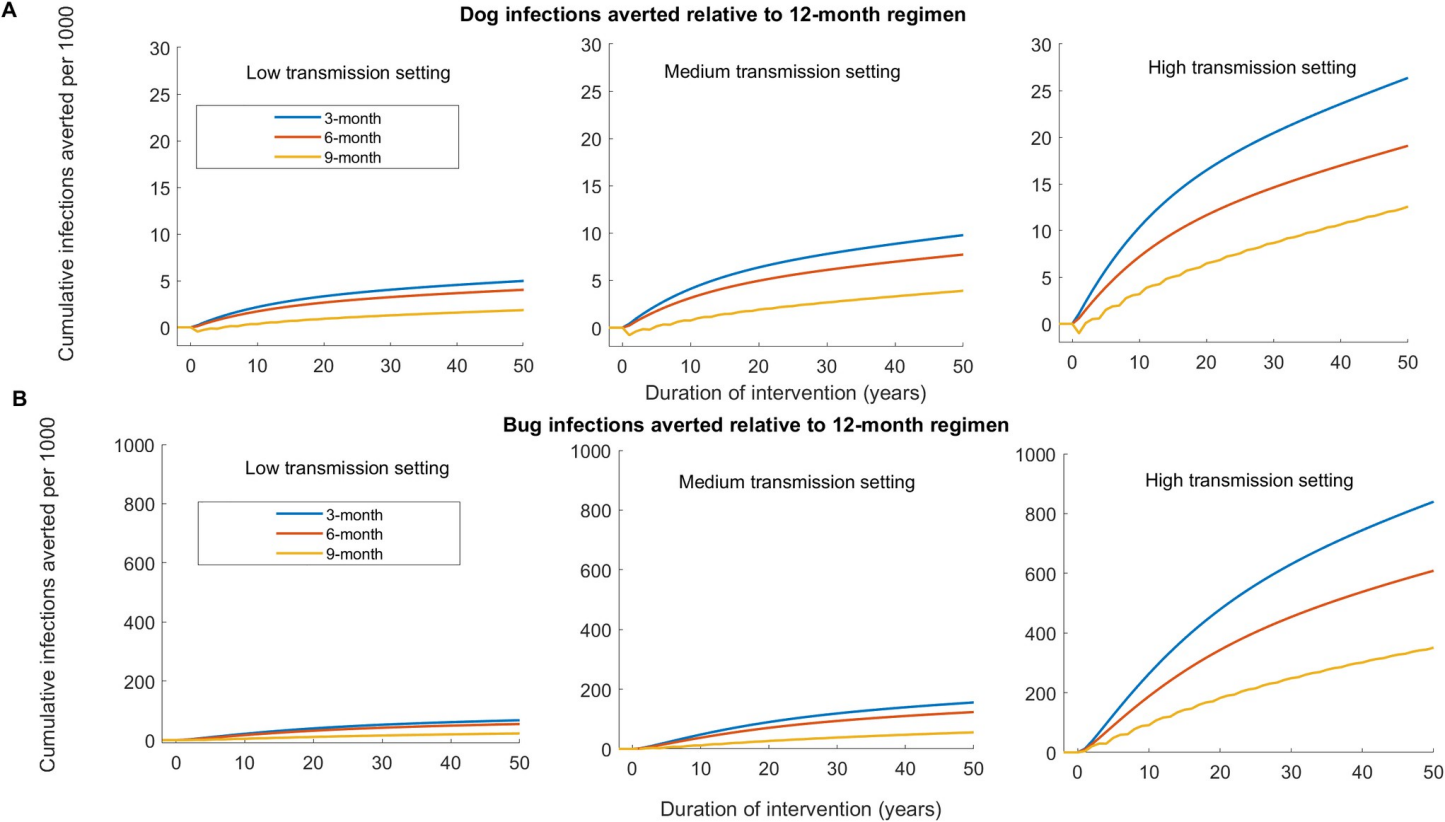
Relative effectiveness of dog treatment regimen for reducing *T*. ***cruzi* infections among dogs and triatomines compared to a 12-month treatment regimen using Model 3.** Effectiveness is computed using the spatially coupled model. (A) Cumulative additional dog infections averted under the 3-month, 6-month, and 9-month regimen relative to the 12-month regimen in the low, medium, and high transmission settings. (B) Cumulative additional triatomine infections averted under the 3-month, 6-month, and 9-month regimen relative to the 12-month regimen in the low, medium, and high transmission settings.

In addition to the single vector-host model, we also consider a spatially coupled vector-host model with triatomines migrating between peridomestic and sylvatic transmission cycle settings. For simplicity, we assumed that a fixed proportion of triatomines in each setting migrate to the other setting annually (migration rate). Similar to the single population model, we observed a rapid decline in vector population density, vector *T*. *cruzi* prevalence, and dog *T*. *cruzi* incidence in all transmission settings (Figs 7, F, and G in S1 Text). Results for the high transmission setting are shown in Fig 7, and those of low and medium transmission settings are shown in Figs F and G in S1 Text, respectively. For instance, after 10 years of treatment in the high transmission setting, bug’s population density was reduced on average by 79.7%, 79.7%, 77.2%, and 72.4% under the 3-month, 6-month, 9-month, and 12-month treatment regimens, respectively (Fig 7A). However, contrary to the single vector-host model, the spatially coupled model showed a sustained prevalence of *T*. *cruzi* among triatomines as high as 5% of the prevalence during the pretreatment period, and dog *T*. *cruzi* incidence was reduced by 90% (Fig 7B). Under the 3-month, 6-month, 9-month, and 12-month treatment regimen, *T*. *cruzi* prevalence among dogs was reduced by 33.2%, 32.7%, 32.1%, and 31.6%, respectively, after five years of repeated treatment, and by 55.9%, 55.3%, 54.5%, and 53.8% after ten years of repeated treatment (Table A in S1 Text). In high transmission settings, we showed that the 3-month treatment regimen was shown to be more effective in reducing *T*. *cruzi* infections in the peridomestic transmission cycle (both dogs and triatomines) than the other three regimens (Fig 8). However, in a low transmission setting, the difference between the 3-month and 6-month regimens was marginal (Fig 8). The effectiveness of treatment regimens for reducing dog infection was shown to increase with transmission intensity; with high transmission setting having the highest reduction and low transmission having the lowest reduction (Fig 8 and Table A in S1 Text).

**Fig 9:**
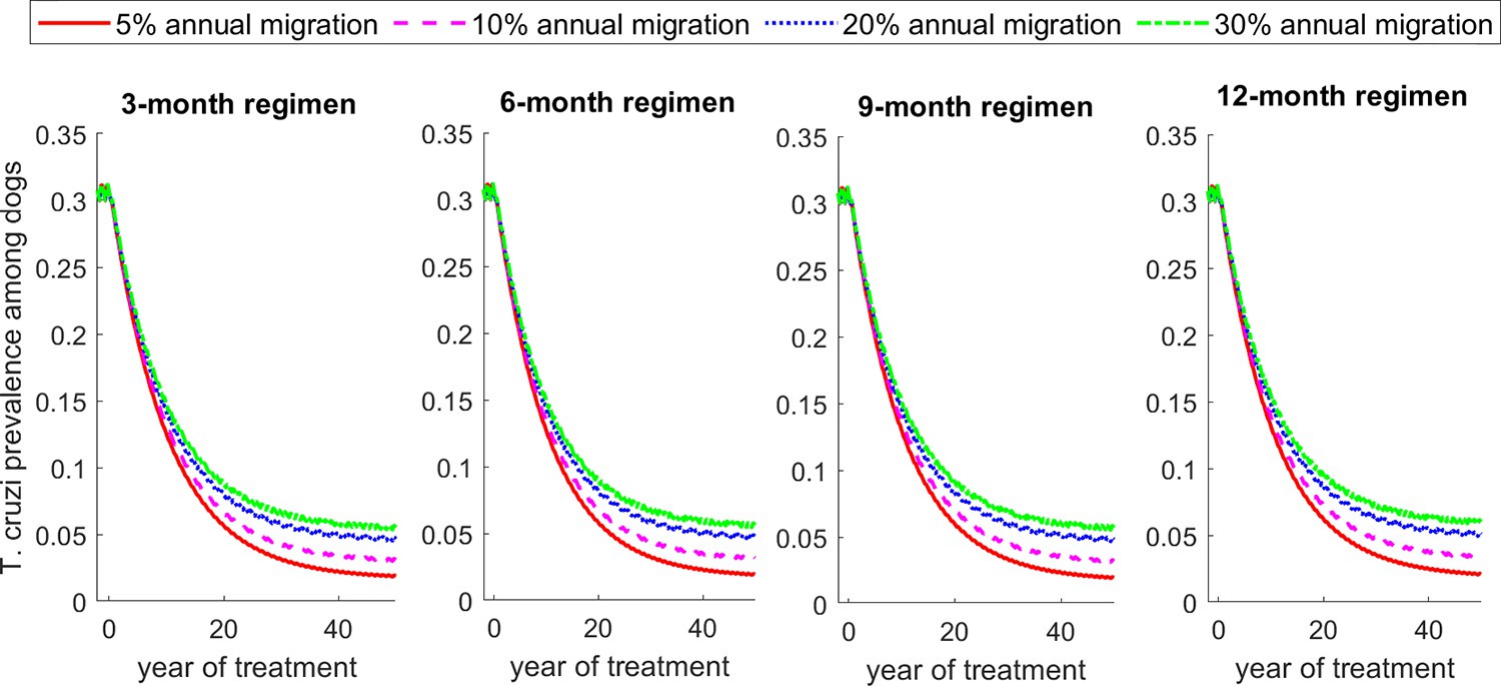
Impact of triatomine migration rate between sylvatic and peridomestic communities on the effectiveness of fluralaner treatment regimens for reducing dog *T*. *cruzi* prevalence.

Finally, we evaluate the impact of triatomine migration on the effectiveness of dog treatment regimens for reducing *T*. *cruzi* infection prevalence in dogs (Fig 9). We show that increased migration rate reduces the effectiveness of fluralaner for all treatment regimens, but the relative reduction of effectiveness is marginal during the first years of treatment (Fig 9).

### Impact of increased consumption of dead triatomines

**Fig 10:**
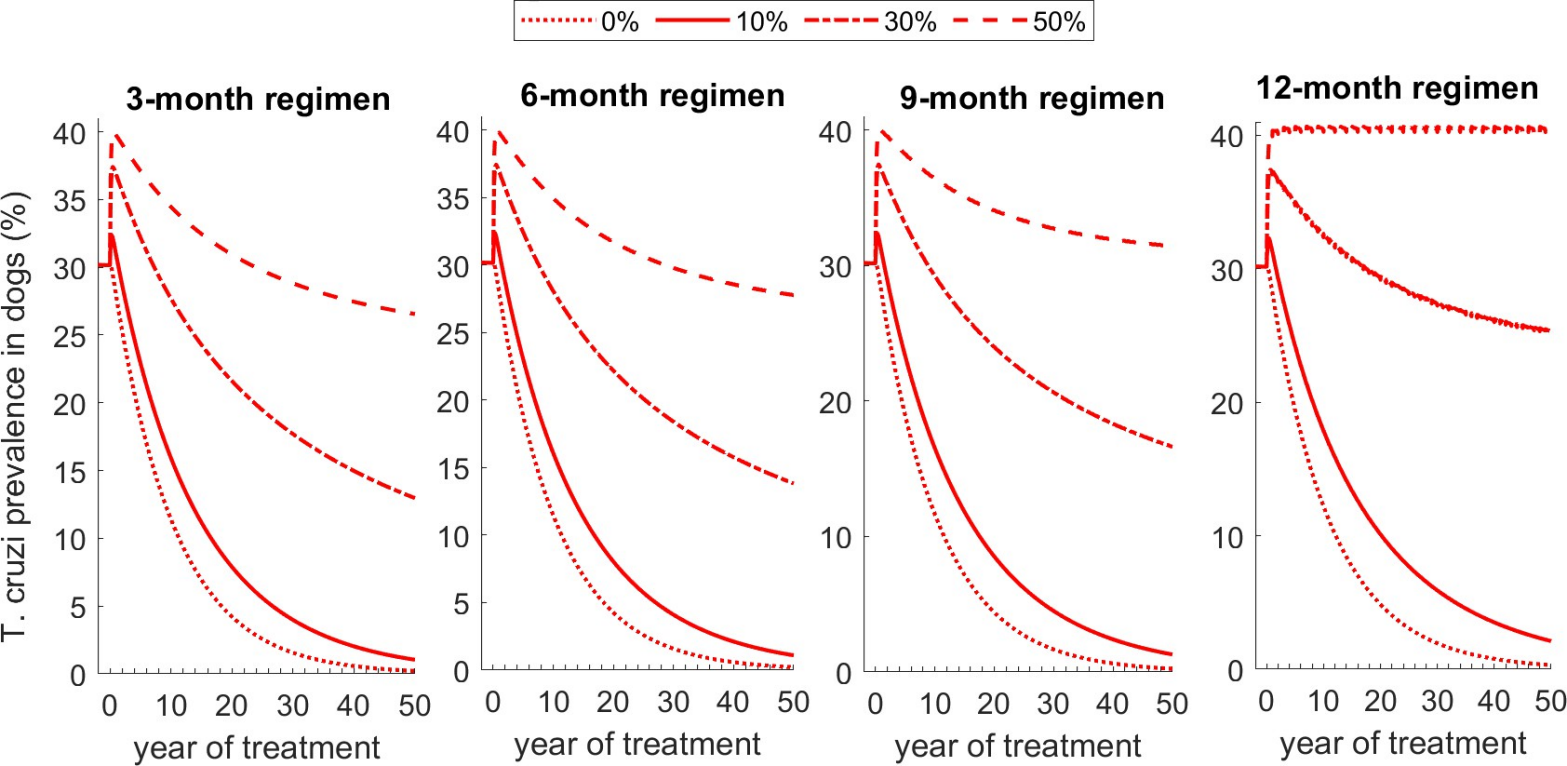
Impact of increased consumption of dead triatomines by dogs on the effectiveness of fluralaner treatment regimens for reducing dog *T*. *cruzi* prevalence. We consider four scenarios: 1) no increased consumption of dead bugs, 2) 10% of bugs killed by fluralaner treatment are eaten by dogs, and 3) 30% of bugs killed by fluralaner treatment are eaten by dogs, and 4) 50% of bugs killed by fluralaner treatment are eaten by dogs.

We show that if increased contacts between dogs and dead triatomines, killed from fluralaner treatment, result in an increased oral consumption of dead triatomines by dogs beyond the baseline number of bugs eaten during pre-treatment period, this could reduce the effectiveness of fluralaner treatment for reducing *T*. *cruzi* infection prevalence in dogs and even potentially increased *T*. *cruzi* infection prevalence. This impact varies with the additional proportion of triatomines eaten by dogs, and the frequency of fluralaner treatment (Fig 10). For example, we show that if 10% of killed triatomines were eaten by dogs, it would result in a small increase of *T*. *cruzi* infection prevalence in dogs during the first year of treatment, followed by a quick and consistent decline of prevalence below pre-treatment level (Fig 10). If 30% of killed triatomines were eaten by dogs, it would result in a substantial increase of *T*. *cruzi* infection prevalence in dogs during the first year of treatment, and prevalence remain above pre-treatment level for five to 20 years following treatment initiation depending on the frequency of treatment (Fig 10). However, *T*. *cruzi* infection prevalence in triatomines is substantially reduced below its pre-treatment level (Fig H in S1 Text). Similar results were observed for all transmission settings, with and without seasonal effects (results not shown here). In all scenarios, the increase in *T*. *cruzi* infection prevalence in dogs occurred during the first year of treatment.

## Discussion

Using compartmental models, we evaluated the population-level impacts of fluralaner treatment on triatomines, and *T*. *cruzi* infection in dogs and triatomines in a peridomestic environment. Across all transmission settings and treatment regimens, fluralaner intervention reduces the triatomine population density. High treatment frequency is always more effective in reducing *T*. *cruzi* infection prevalence in the dog population. In low transmission settings, this difference may be negligible, indicating that less frequent treatment in these settings may be sufficient to reduce *T*. *cruzi* infections in dogs and triatomines (Figs A, C, and F in S1 Text). These results show fluralaner treatment can reduce triatomine populations and *T*. *cruzi* infection in peridomestic environments and reduce the risk of Chagas disease in dogs housed in peridomestic environments. These results agree with those of a recent placebo-controlled study on the effect of fluralaner on the control of triatomines *T*. *cruzi* infection [81]. However, similar to Rokhsar at al. [34], we show that if increased triatomine mortality from dogs’ treatment resulted in a significant increase in dog consumption of dead triatomines relative to the pre-treatment era (e.g. more than 10% of killed triatomines are eaten by dogs in surplus of the number of bugs generally eaten by dogs during the pre-treatment era), fluralaner treatment may have a counterproductive effect, resulting in an increased prevalence of *T*. *cruzi* infections in dogs at least during the first years of treatment (Fig 10).

In previous studies, dogs that were housed with triatomines in a closed environment consumed 12–27% of the live triatomines present, even without fluralaner treatment [82,83]. Thus, in a peridomestic setting, the oral transmission route may be an important route of infection for dogs, and if fluralaner increases the availability of dead and infected triatomines around dogs, the oral route may be even more important as a driver of canine *T*. *cruzi* transmission. However, since insects are neither a significant portion of a dog’s diet nor are dogs’ insectivores, we don’t anticipate the increased mortality of triatomines would result in a substantial increase in consumption of triatomines by dogs. Moreover, triatomines’ death by xenointoxication may occur at least 24 hours after feeding [32], which allows triatomines enough time to return to their refuge before dying rather than dying instantaneously after biting treated dogs. Therefore, fluralaner treatment of dogs may not necessarily increase dog consumption of triatomines compared to the number of bugs eaten during the pre-treatment period. To provide a more accurate estimate of the potential impact of systemic insecticide use on *T*. *cruzi* infection in dogs, future studies should provide a better understanding of dogs’ bug eating behavior, the impact of systemic insecticide use on contact between dogs and death triatomines, and the impact of increase triatomine mortality on dogs’ triatomine eating behavior. Future studies should also investigate triatomine *T*. *cruzi* infectivity and duration of infection after death.

The degree to which these changes are observed in our models varies based on the regimen of treatment, transmission settings, impact of seasonality of triatomine population dynamics, and triatomine migration rate. In the presence of seasonality and high triatomine migration rate between peridomestic and sylvatic cycles, treating dogs more frequently becomes more important in higher transmission areas. In low and medium transmission areas, 3-month, and 6-month treatment regimens were shown to be highly or equally effective in reducing *T*. *cruzi* infection incidence among dogs. In the presence of triatomine migration between peridomestic and sylvatic settings, the difference in effectiveness between the 3-month and 6-month regimens was more pronounced. As triatomine population and *T*. *cruzi* infections in the peridomestic setting are replenished through migration, more frequent dog treatment becomes more effective for disease control, especially in medium and high transmission settings (Fig 9). Thus, in low-transmission settings, treating every 6 months may be sufficient to control *T*. *cruzi* infections in dogs and triatomines, which could be a cost-saving measure.

Seasonality introduces oscillations into triatomine population sizes, leading to changes throughout the year in triatomine movement, interactions with dogs, and risk of infection [36]. By ignoring seasonality, models are likely to overestimate *T*. *cruzi* transmission risk during triatomine low activity season and underestimate the risk during high activity season. This is likely to impact the effectiveness of low frequency treatment, such as 9-month and 12-month treatment regimens, especially if treatment is not administered at the start of the triatomine high activity season (early Spring). Specifically, for the 9-month treatment regimen, which may or may not result in treatment being given just prior to the peak triatomine season, seasonality has the potential to decrease the efficacy of treatment (Fig 6).

As a neglected tropical disease, there is limited and sparse spatio-temporal data on the spread of Chagas disease, especially in the peridomestic and sylvatic transmission cycles modeled here. This substantially limits the available parameter values or the ability to estimate them through model fitting to data. For example, the impact of seasonality on the triatomine life cycle as well as their migration rates between sylvatic and peridomestic environments have been shown to be important factors to disease transmission, but are not well-characterized. To address this limitation, we used parameter values widely used in the Chagas disease modeling literature as well as available data on monthly variation of triatomine host biting [76] and dispersal [77] to inform the functional form of seasonality of contact rate between triatomines and dogs and triatomine migration between sylvatic and peridomestic transmission settings. We also conducted sensitivity analyses on our migration and transmission rates. We assume our model is in an endemic setting and is at equilibrium; however, our parameter estimates are calibrated to single data points, which may not accurately reflect underlying transmission dynamics. While this is a common technique in modeling studies, it does not consider that prevalence values change over time, even in endemic settings. Future studies characterizing time series data can be used to fit the model and better capture underlying transmission dynamics and more accurate estimates of epidemiological parameters. We assume dogs live on average 10 years, without taking into consideration disease-induced death or other dog age-related factors. Future work should consider an age-structured model for dogs and the impact of Chagas disease on dog mortality. In our models, we only assess fluralaner as an intervention. Future modeling approaches could investigate the addition of other recommended integrated pest management techniques, such as removing woody debris and harborage for wild reservoir mammals, turning off exterior lights that attract dispersing adult triatomines, and improving kennels and houses to reduce triatomine entrance and colonization, which may further reduce *T*. *cruzi* transmission [7]. However, in our experience working at several large dog’s kennels in south Texas where various integrated triatomine management techniques are practiced, canine incidence was high across multiple kennels [23].

Our study suggests that all dogs, including peridomestic dogs, will have to be treated every three to six months for at least five years to control the spread of Chagas disease in endemic communities. In the southern United States, where triatomines and canine Chagas disease are endemic, such control strategies are feasible as peridomestic dogs are mostly kennels and working dogs. However, in Latin America, peridomestic dogs in Chagas disease highly endemic communities are mostly stray dogs. In these settings treating all peridomestic dogs every three to six months for at least five years may be extremely challenging and would likely require significant public health and financial resources. Future studies should investigate the feasibility, cost-effectiveness, and budget impact analyses of these control strategies in different chagas endemic communities in Latin America.

Our models offer valuable insight into transmission dynamics in the *T*. *cruzi* peridomestic transmission cycle and test the outcomes of implementation of xenointoxication-based control in this setting. We show that canine and triatomine *T*. *cruzi* infections may be substantially averted with the routine use of systemic insecticides. In low and medium transmission environments, less frequent treatment may be sufficient to reduce *T*. *cruzi* in dogs and triatomines when compared to high transmission environments. However, the use of systemic insecticides may potentially increase canine *T*. *cruzi* infections if increased triatomine mortality results in a substantial increase in dog’s oral consumption of dead triatomines. For this reason, it is paramount to better understand dog’s consumption behavior of dead triatomines in the presence and absence of systemic insecticide use before recommending large-scale and routine use of fluralaner in peridomestic environments.

## Supporting information

S1 Text Supplementary information containing 1. model description of the spatially coupled model. 2. **Fig A.** Effectiveness of systemic insecticide treatment of dogs with fluralaner for the control of canine Chagas in a low transmission setting using Model. 3. **Fig B.** Effectiveness of systemic insecticide treatment of dogs with fluralaner for the control of canine Chagas in a medium transmission setting using Model 1. 4. **Fig C.** Effectiveness of systemic insecticide treatment of dogs with fluralaner for the control of canine Chagas in a low transmission setting using Model 2. 5. **Fig D.** Effectiveness of systemic insecticide treatment of dogs with fluralaner for the control of canine Chagas in a medium transmission setting using Model 2. 6. **Fig E.** Comparing effectiveness results for *ε* equals to 0.25 vs 0.5. Relative effectiveness of dog treatment regimen for reducing *T*. *cruzi* infections among dogs and triatomines compared to a 12-month treatment regimen using Model 2. 7. **Fig F.** Effectiveness of systemic insecticide treatment of dogs with fluralaner for the control of canine Chagas in a low transmission setting using Model 3. 8. **Fig G**. Effectiveness of systemic insecticide treatment of dogs with fluralaner for the control of canine Chagas in a medium transmission setting using Model 3. 9. **Fig H.** Dynamics of *Trypanosoma cruzi* prevalence in triatomines and dogs under the 3-month, 6-month, 9-month, and 12-month treatment regimen in high, medium, and low transmission settings under the assumption that 50% of triatomines killed by fluralaner treatment are eaten by dogs in addition to the baseline number of triatomines eaten by dogs (number of triatomines eaten during the pre-treatment period). 10. **Table A.** Reduction of *T*. *cruzi* prevalence among a dog population given different treatment regimens of a systemic insecticide (fluralaner) in various transmission settings.
(DOCX)

S1 MatLab-codes Zip file containing MatLab codes of the models. These files require access to the MatLab software to be opened and run.
(ZIP)
