# Supplementary material for: Effectiveness of fluralaner treatment regimens for the control of canine Chagas disease: A mathematical modeling study
Source: PLoS Negl Trop Dis. 2023 Jan 24;17(1):e0011084. doi: 10.1371/journal.pntd.0011084 (PMC9897538; doi:10.1371/journal.pntd.0011084)
Supplement: S1 Text — model description of the spatially coupled model. 2. Fig A. Effectiveness of systemic insecticide treatment of dogs with fluralaner for the control of canine Chagas in a low transmission setting using Model. 3. Fig B. Effectiveness of systemic insecticide treatment of dogs with fluralaner for the control of canine Chagas in a medium transmission setting using Model 1. 4. Fig C. Effectiveness of systemic insecticide treatment of dogs with fluralaner for the control of canine Chagas in a low transmission setting using Model 2. 5. Fig D. Effectiveness of systemic insecticide treatment of dogs with fluralaner for the control of canine Chagas in a medium transmission setting using Model 2. 6. Fig E. Comparing effectiveness results for ε equals to 0.25 vs 0.5. Relative effectiveness of dog treatment regimen for reducing T. cruzi infections among dogs and triatomines compared to a 12-month treatment regimen using Model 2. 7. Fig F. Effectiveness of systemic insecticide treatment of dogs with fluralaner for the control of canine Chagas in a low transmission setting using Model 3. 8. Fig G. Effectiveness of systemic insecticide treatment of dogs with fluralaner for the control of canine Chagas in a medium transmission setting using Model 3. 9. Fig H. Dynamics of Trypanosoma cruzi prevalence in triatomines and dogs under the 3-month, 6-month, 9-month, and 12-month treatment regimen in high, medium, and low transmission settings under the assumption that 50% of triatomines killed by fluralaner treatment are eaten by dogs in addition to the baseline number of triatomines eaten by dogs (number of triatomines eaten during the pre-treatment period). 10. Table A. Reduction of T. cruzi prevalence among a dog population given different treatment regimens of a systemic insecticide (fluralaner) in various transmission settings. (DOCX) [file pntd.0011084.s001.docx]

**S1 Text**

Effectiveness of fluralaner treatment regimens for the control of canine Chagas disease: A mathematical modeling study

Edem Fiatsonu^1*^, Rachel E Busselman^1*^, Gabriel L Hamer^2^, Sarah A Hamer^1^, Martial L Ndeffo-Mbah^1,3@^

1. Department of Veterinary Integrative Biosciences, College of Veterinary Medicine and Biomedical Sciences, Texas A&M University, Texas, USA
2. Department of Entomology, College of Agriculture and Life Sciences, Texas A&M University, Texas, USA
3. Department of Epidemiology and Biostatistics, School of Public Health, Texas A&M University, Texas, USA

**Model description**

## Spatially coupled vector-host populations with impact of seasonality on triatomines

Our model extends the dynamics of Chagas disease in both peridomestic and sylvatic habitats where the population structure here remains the same as the single vector-host population setting for simplicity. However, in the sylvatic setting, $E_{w}$ is the total number of triatomine eggs, and $Y_{w}$ is the number of nymphs. We assumed adult triatomines could be susceptible $S_{bw}$ (not infected with the *T. cruzi* and have the ability to become infected) or infectious $I_{bw}$ (infected with *T. cruzi* and have the ability to transmit to vertebrate hosts). The total adult population density of triatomines in the wildlife environment is $N_{bw}=S_{bw}+ I_{bw}$. The host’s population $N_{w}$ in the sylvatic habitat is assumed to be constant; that is, birth rate is the same as the death rate. Each host could be in one of two mutually exclusive disease states: susceptible $S_{w}$population (not infected with *T. cruzi* and able to become infected), or infectious $I_{w}$ population (infected with *T. cruzi* and able to transmit), where *T. cruzi* transmission is primarily by host-triatomine contacts. The movement rate of adult triatomines between these two habitats is a function m(t) at time t. The function takes the form

$\eta(t)=\eta^{0}m(t)$ and $\zeta(t)=\zeta^{0}m(t)$ with $\int_{0}^{1} m(t)dt=1$ informed from [77]. The annual transmission rate $\beta_{vw}$ of vector in the wildlife and the annual host transmission in the wild are $\beta_{vw}={\beta_{vw}}^{0}w(t)$and $\beta_{hw}={\beta_{hw}}^{0}w(t)$ with $\int_{0}^{1} w(t)dt=1$

also informed from [76]. The last two terms in the equations 1c, 1d, 1e, 1j and 1k describe the net movement rate of triatomines between these two habitats, where $\eta(t)$ is the movement rate from the peridomestic to sylvatic habitat and $\zeta(t)$ is the movement rate from the sylvatic to peridomestic habitat. Here, we assumed an equal movement rate of adult triatomines between the two habitats. The mortality rate $\delta_{b}$ of the adult triatomines and mortality rate $\delta_{w}$ for other hosts in the sylvatic habitat is assumed to be the same as that in the peridomestic habitat and single vector-host setting.

$\frac{dE}{dt}=\lambda N_{b}-\tau E-\delta_{e}E$ 1a

$\frac{dY}{dt}=\tau E(1-\frac{Y+ N_{b}}{\kappa})-\gamma_{y}(t)Y-\delta_{y}Y$ 1b

$\frac{dS_{b1}}{dt}=\phi\gamma_{y}(t)Y-\beta_{v}{(t)S}_{b1}\frac{I_{h}}{N_{h}}-\delta_{b}S_{b1}-\eta(t)S_{b1}+\zeta(t)\phi S_{bw}$ 1c

$\frac{dI_{b1}}{dt}=\beta_{v}(t)S_{b1}\frac{I_{h}}{N_{h}}-\delta_{b}I_{b1}-\eta(t)I_{b1}+\zeta(t)I_{bw}$ 1d

$\frac{dS_{b2}}{dt}=(1-\phi{)\gamma}_{y}(t)Y-\delta_{b}S_{b2}-\eta(t)S_{b2}+\zeta(t)(1-\phi)S_{bw}$ 1e

$\frac{dS_{h}}{dt}=\delta_{d}N_{h}-\beta_{h}(t)S_{h}\frac{I_{b1}}{N_{b1}}-\delta_{d}S_{h}$ 1f

$\frac{dI_{h}}{dt}=\beta_{h}(t)S_{h}\frac{I_{b1}}{N_{b1}}-\delta_{d}I_{h}$ 1g

$\frac{dE_{w}}{dt}=\lambda N_{b}-\tau E_{w}-\delta_{e}E_{w}$ 1h

$\frac{dY_{w}}{dt}=\tau E_{w}(1-\frac{Y_{w}+ N_{b}}{\kappa})-\gamma_{y}(t)Y_{w}-\delta_{y}Y_{w}$ 1i

$\frac{dS_{bw}}{dt}=\gamma_{y}(t)Y_{w}-\beta_{vw}(t)S_{bw}\frac{I_{w}}{N_{w}}-\delta_{b}S_{bw}+\eta(t)(S_{b1}{+S}_{b2})-\zeta(t)S_{bw}$ 1j

$\frac{dI_{bw}}{dt}=\beta_{vw}(t)S_{bw}\frac{I_{w}}{N_{w}}-\delta_{b}I_{bw}+\eta(t)S_{b1}-\zeta(t)I_{bw}$ 1k

$\frac{dS_{w}}{dt}=\delta_{w}(S_{w}+I_{w})-\beta_{hw}(t)\frac{I_{bw}S_{w}}{(S_{bw}+I_{bw})}-\delta_{w}S_{w}$ 1l

$\frac{dI_{w}}{dt}=\beta_{hw}(t)\frac{I_{bw}S_{w}}{(S_{bw}+I_{bw})}-\delta_{w}I_{w}$ 1m

**Supplementary Figures**


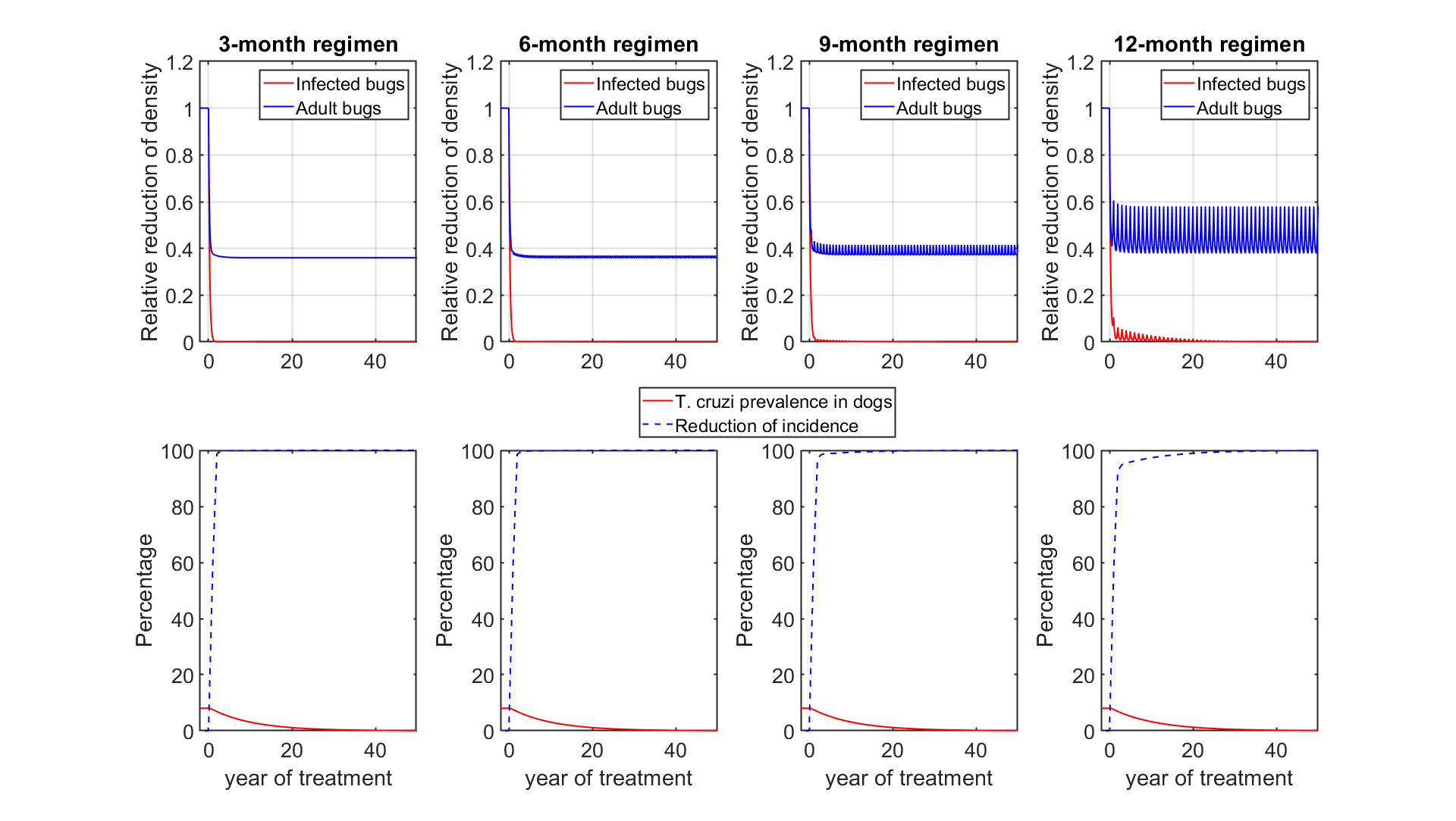


**Fig A**. Effectiveness of systemic insecticide treatment of dogs with fluralaner for the control of canine Chagas in a low transmission setting using Model 1. (A) Reduction of total population density and *T. cruzi* infection in triatomines, (B) Reduction of *T. cruzi* infection prevalence and incidence in dogs. Effectiveness is evaluated using the single vector-host model without seasonality.


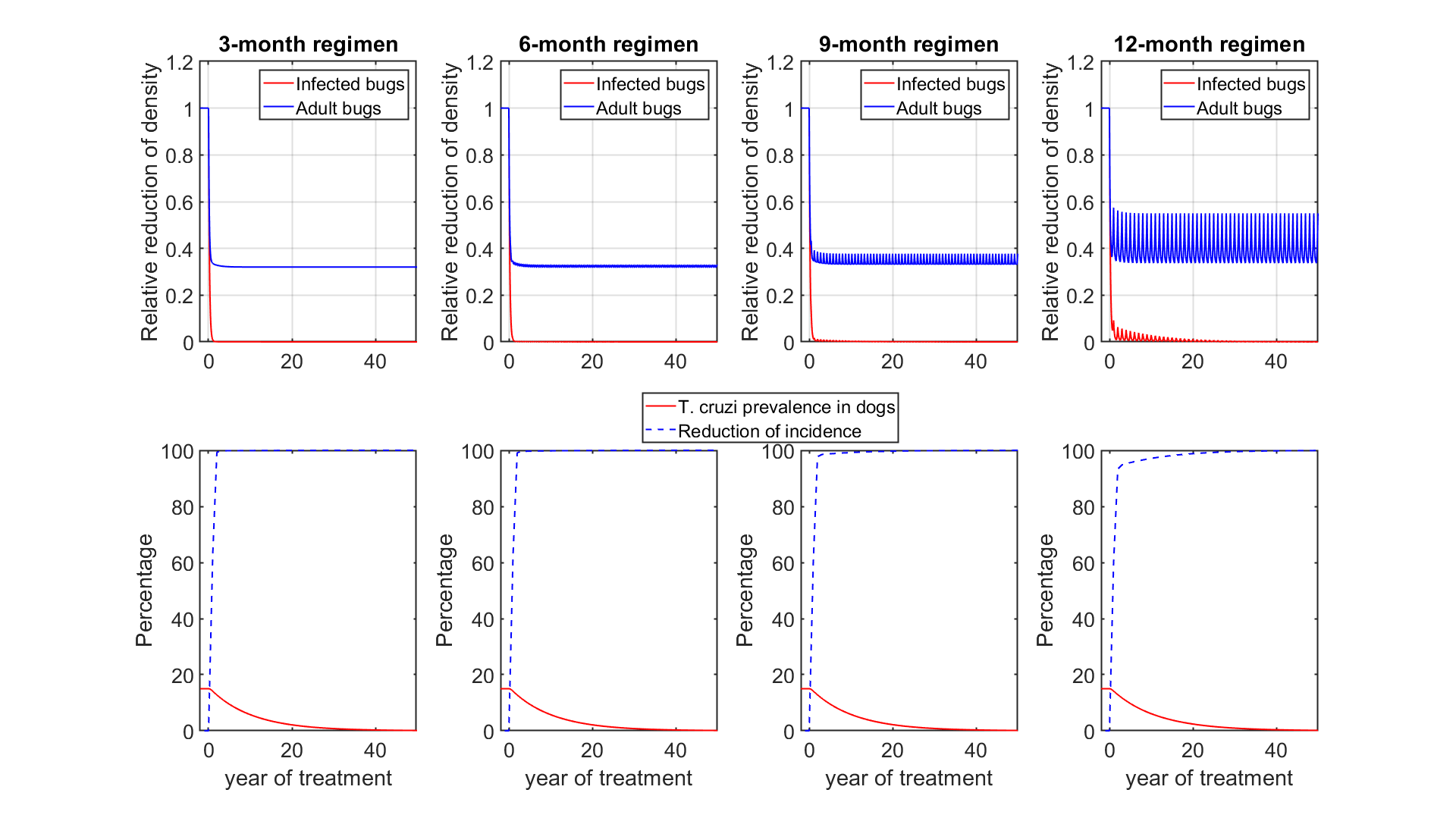


**Fig B.** Effectiveness of systemic insecticide treatment of dogs with fluralaner for the control of canine Chagas in a medium transmission setting using Model 1. (A) Reduction of total population density and *T. cruzi* infection in triatomines, (B) Reduction of *T. cruzi* infection prevalence and incidence in dogs. Effectiveness is evaluated using the single vector-host model without seasonality.


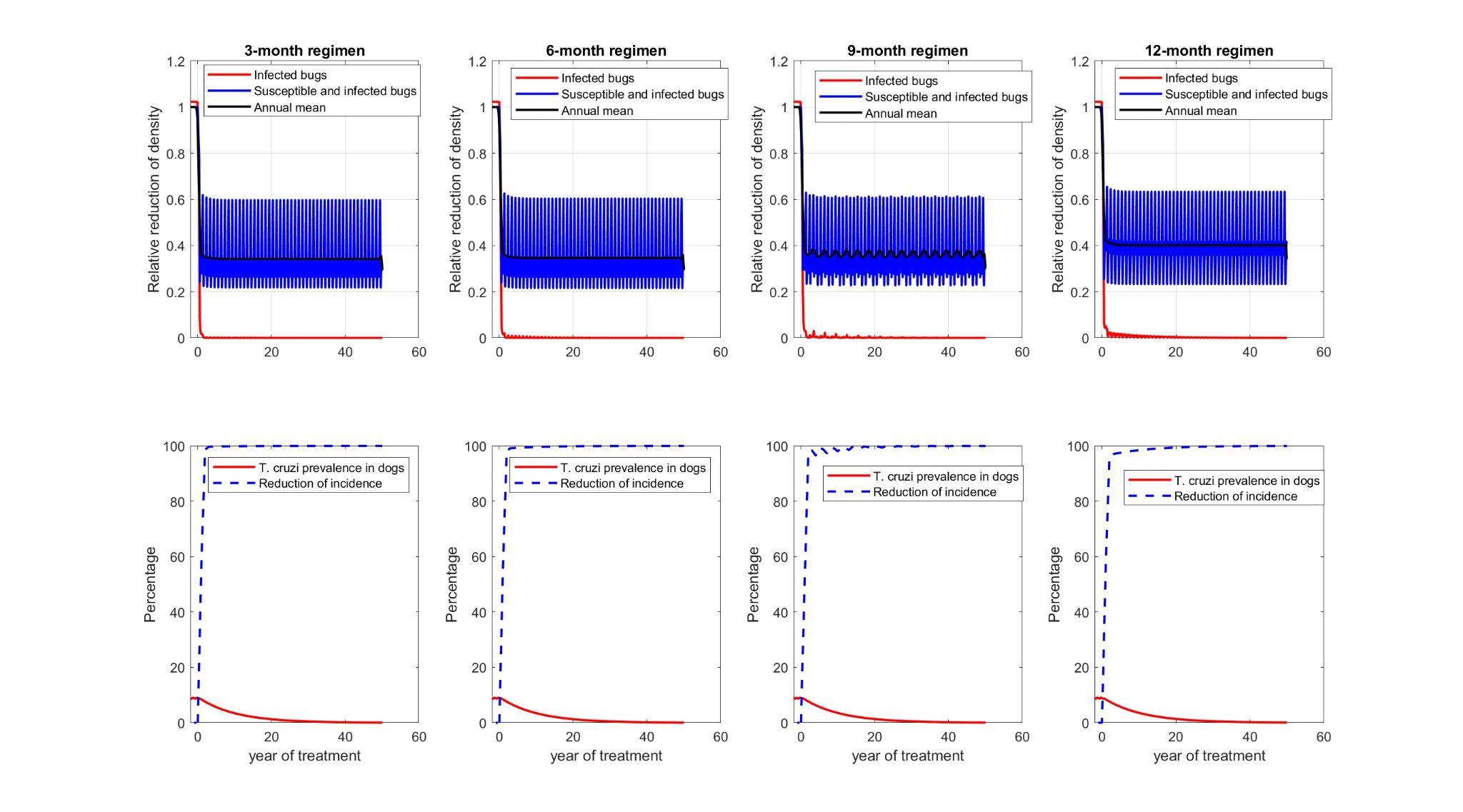


**Fig C.** Effectiveness of systemic insecticide treatment of dogs with fluralaner for the control of canine Chagas in a low transmission setting using Model 2. (A) Reduction of total population density and *T. cruzi* infection in triatomines, (B) Reduction of *T. cruzi* infection prevalence and incidence in dogs. Effectiveness is evaluated using the single vector-host model with seasonality.


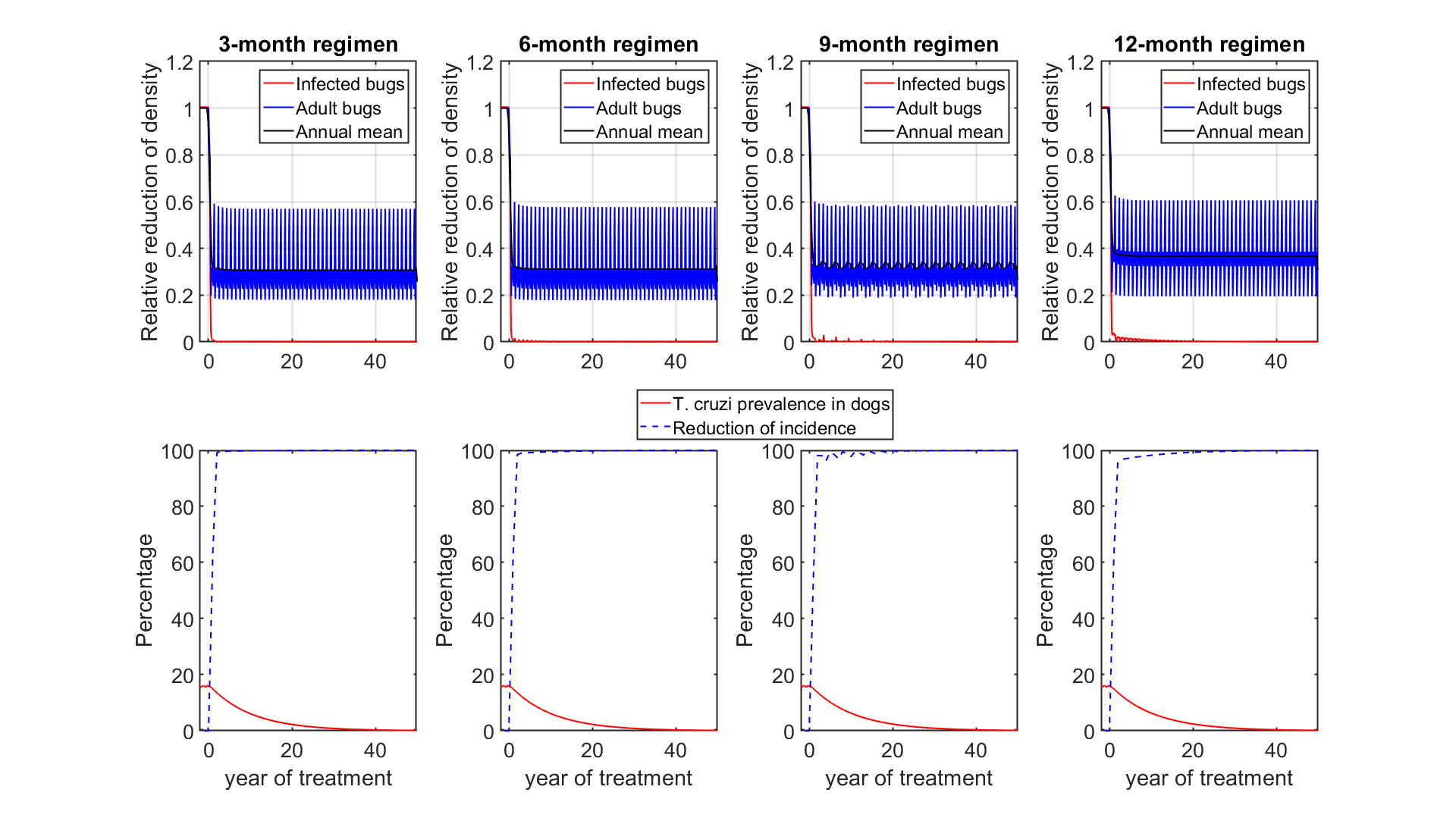


**Fig D.** Effectiveness of systemic insecticide treatment of dogs with fluralaner for the control of canine Chagas in a medium transmission setting using Model 2. (A) Reduction of total population density and *T. cruzi* infection in triatomines, (B) Reduction of *T. cruzi* infection prevalence and incidence in dogs. Effectiveness is evaluated using the single vector-host model with seasonality.


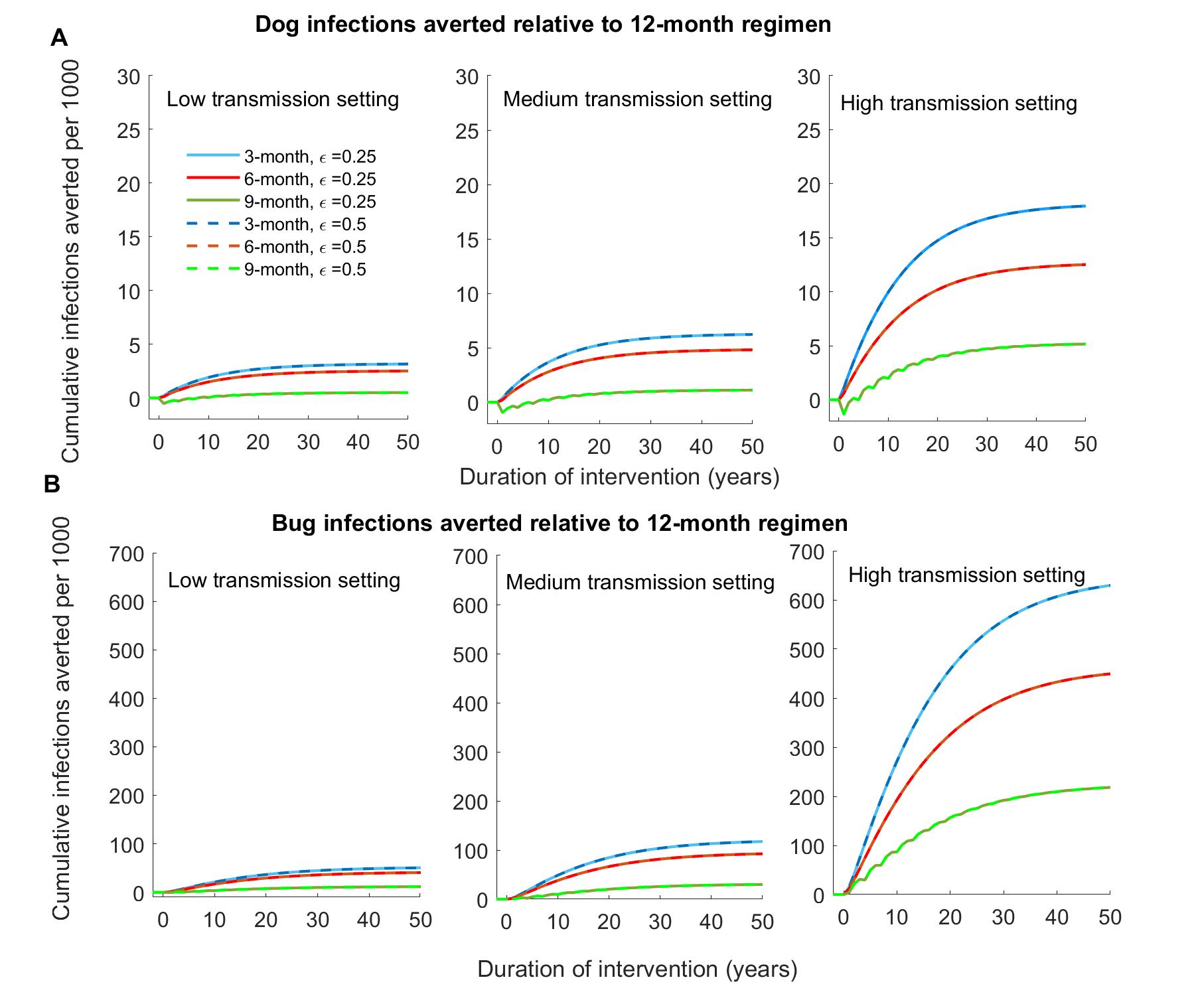


**Fig E.** Comparing effectiveness results for $\varepsilon$ equals to 0.25 vs 0.5. Relative effectiveness of dog treatment regimen for reducing *T. cruzi* infections among dogs and triatomines compared to a 12-month treatment regimen using Model 2. (A) Cumulative additional dog infections averted under the 3-month, 6-month, and 9-month regimen relative to the 12-month regimen in the low, medium, and high transmission settings. (B) Cumulative additional triatomine infections averted under the 3-month, 6-month, and 9-month regimen relative to the 12-month regimen in the low, medium, and high transmission settings.


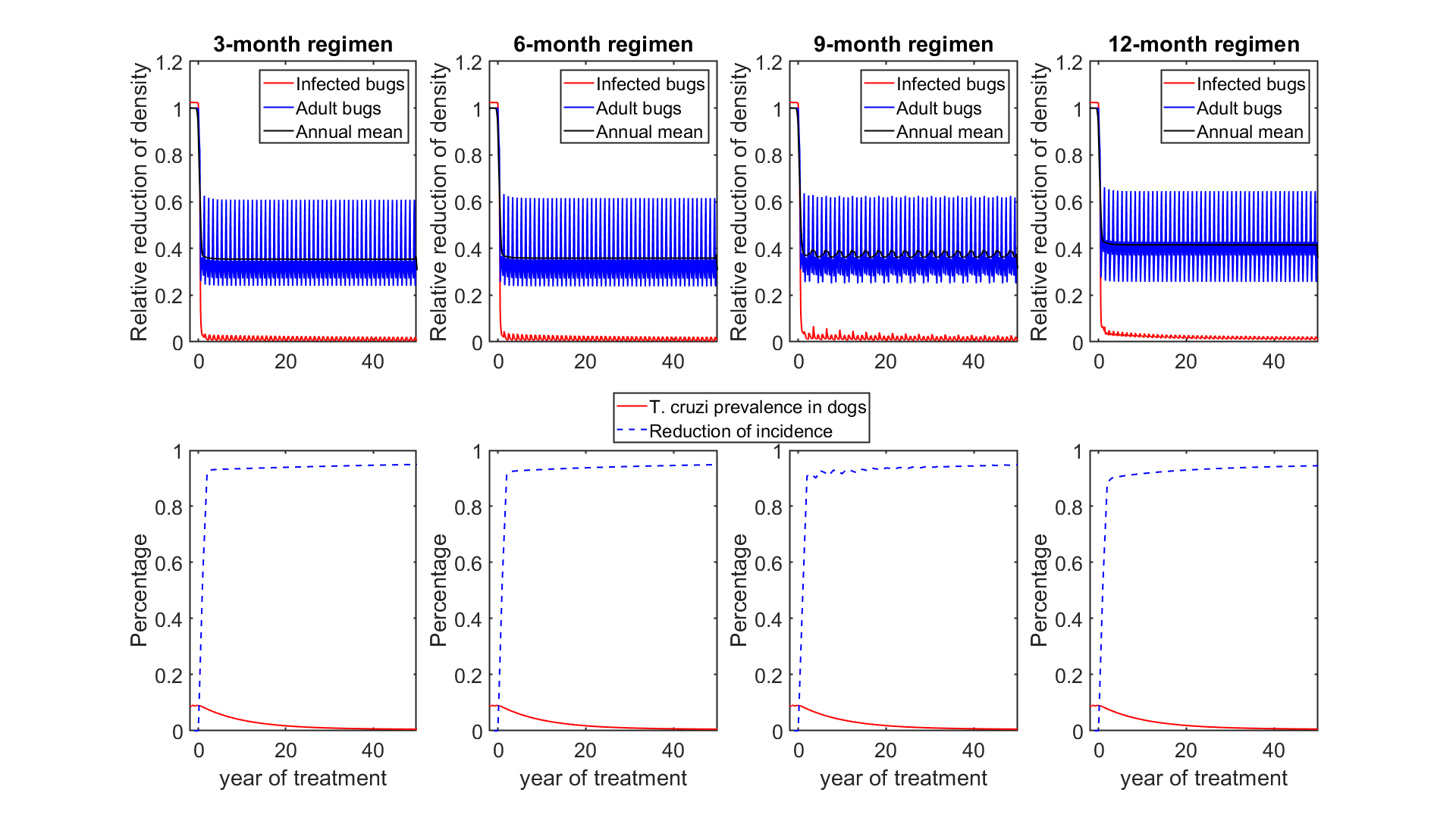


**Fig F**. Effectiveness of systemic insecticide treatment of dogs with fluralaner for the control of canine Chagas in a low transmission setting using Model 3. (A) Reduction of total population density and *T. cruzi* infection in triatomines, (B) Reduction of *T. cruzi* infection prevalence and incidence in dogs. Effectiveness is evaluated using the spatially coupled model.


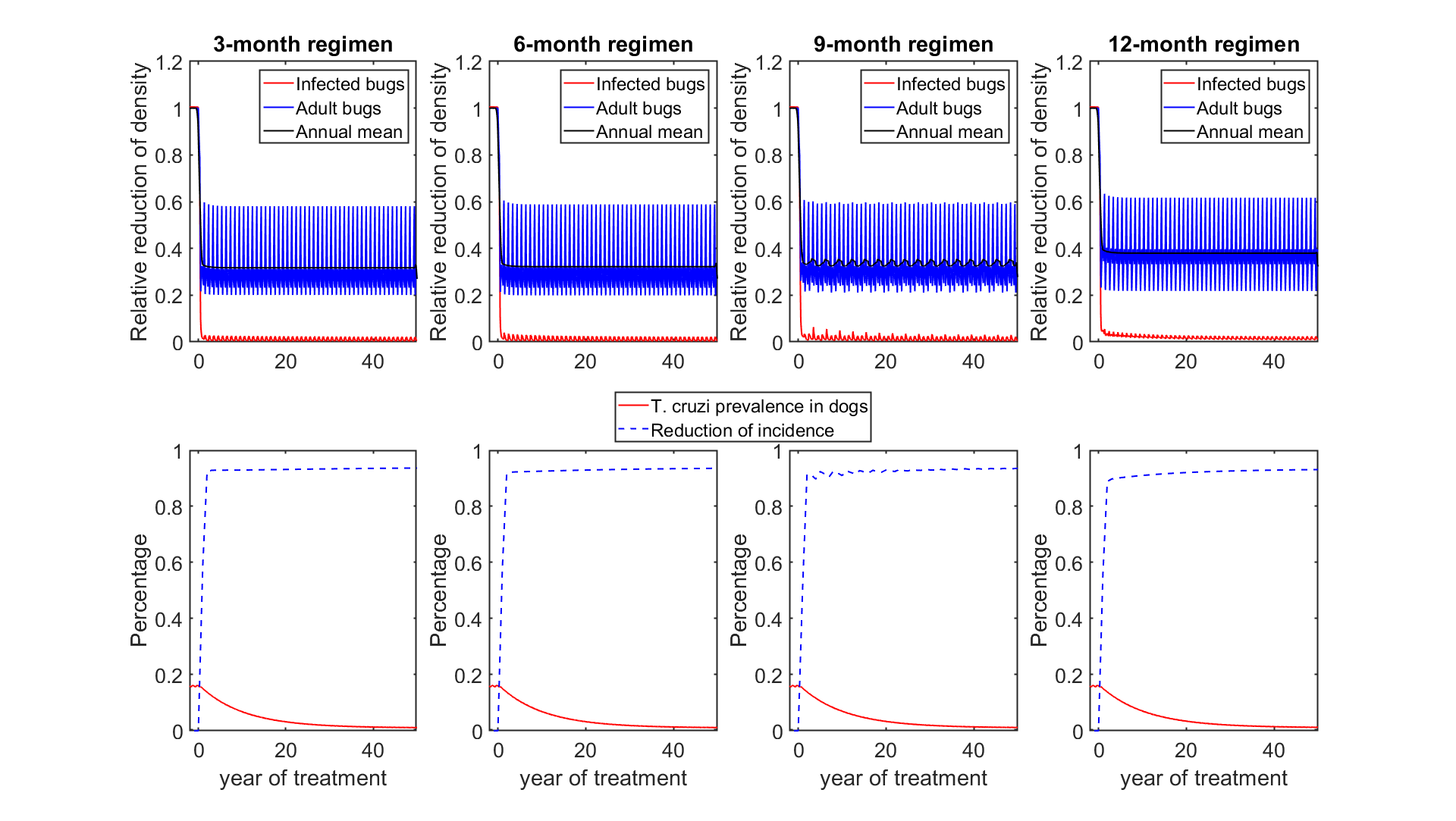


**Fig G**. Effectiveness of systemic insecticide treatment of dogs with fluralaner for the control of canine Chagas in a medium transmission setting using Model 3. (A) Reduction of total population density and *T. cruzi* infection in triatomines, (B) Reduction of *T. cruzi* infection prevalence and incidence in dogs. Effectiveness is evaluated using the spatially coupled model.


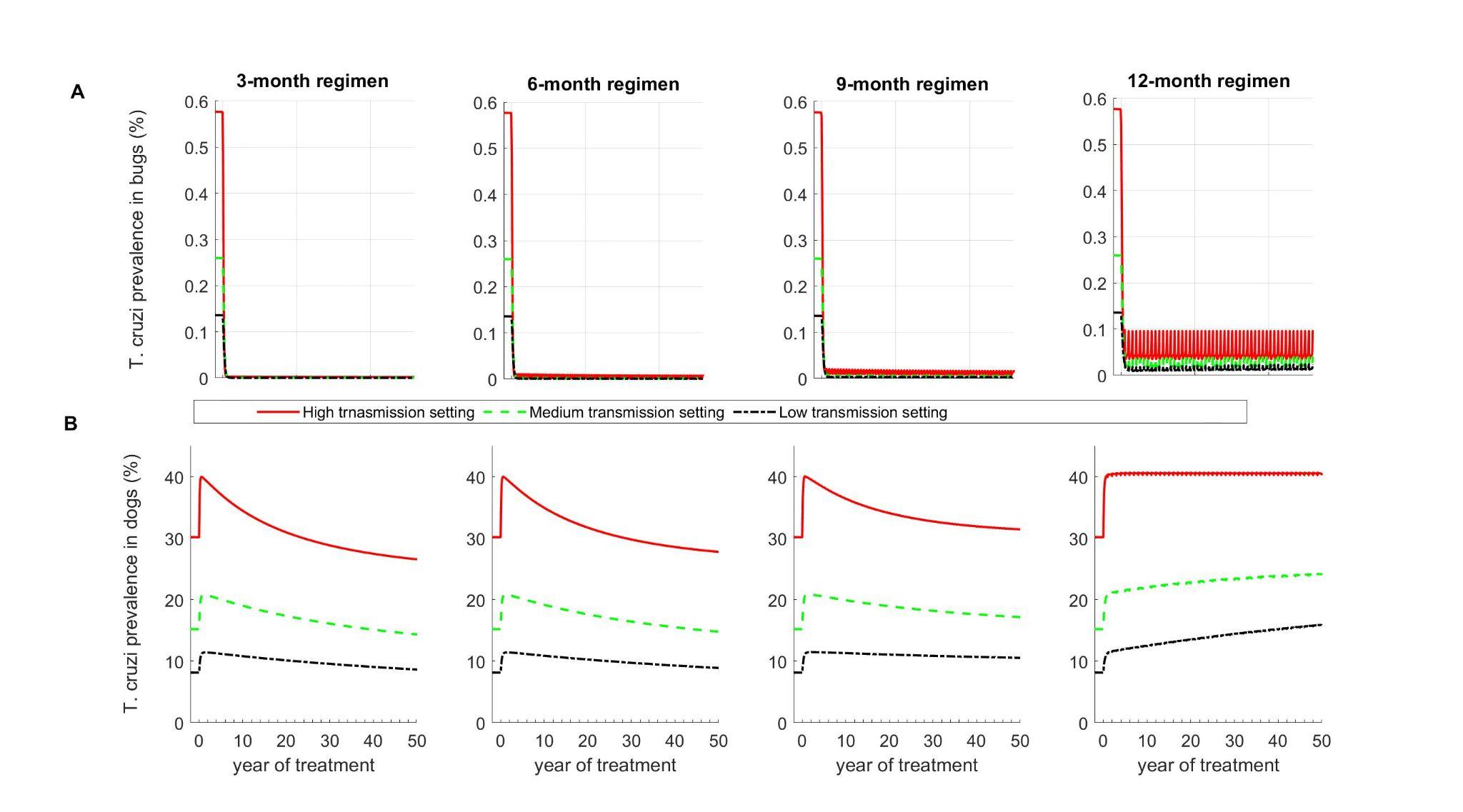

**Fig H.** Dynamics of *Trypanosoma cruzi* prevalence in triatomines and dogs under the 3-month, 6-month, 9-month, and 12-month treatment regimen in high, medium, and low transmission settings under the assumption that 50% of triatomines killed by fluralaner treatment are eaten by dogs in addition to the baseline number of triatomines eaten by dogs (number of triatomines eaten during the pre-treatment period).

**Table A.** Reduction of *T. cruzi* prevalence among a dog population given different treatment regimens of a systemic insecticide (fluralaner) in various transmission settings.

| Regimen | Years of treatment | High transmission | Medium transmission | Low transmission |
| --- | --- | --- | --- | --- |
| Without seasonality | | | | |
| 3-month | 5 | 37.70 % | 36.93 % | 36.50 % |
|  | 10 | 63.13 % | 61.67 % | 61.38 % |
| 6-month | 5 | 37.57 % | 36.80 % | 36.38 % |
|  | 10 | 61.97 % | 61.60 % | 61.37 % |
| 9-month | 5 | 37.10 % | 36.33 % | 35.88 % |
|  | 10 | 61.37 % | 61.60 % | 60.75 % |
| 12-month | 5 | 35.70 % | 35.13 % | 34.75 % |
|  | 10 | 59.37 % | 59.33 % | 59.13 % |
| With seasonality | | | | |
| 3-month | 5 | 36.20 % | 33.00 % | 29.50 % |
|  | 10 | 61.13 % | 59.27 % | 57.13 % |
| 6-month | 5 | 35.73 % | 32.73 % | 29.25 % |
|  | 10 | 60.50 % | 58.93 % | 56.88 % |
| 9-month | 5 | 35.03 % | 31.87 % | 28.25 % |
|  | 10 | 59.60 % | 58.00 % | 55.88 % |
| 12-month | 5 | 34.70 % | 31.87 % | 28.38 % |
|  | 10 | 59.13 % | 57.80 % | 55.75 % |
| Spatial coupling | | | | |
| 3-month | 5 | 33.20 % | 30.07 % | 26.38 % |
|  | 10 | 55.97 % | 54.60 % | 52.38 % |
| 6-month | 5 | 32.73 % | 29.80 % | 26.00 % |
|  | 10 | 55.33 % | 54.27 % | 52.00 % |
| 9-month | 5 | 32.13 % | 29.00 % | 25.25 % |
|  | 10 | 54.57 % | 53.00 % | 51.13 % |
| 12-month | 5 | 31.67 % | 28.87 % | 25.13 % |
|  | 10 | 53.87 % | 53.00 % | 50.75 % |
